# Chromatin remodeler Dmp18 regulates apoptosis by controlling H2Av incorporation in *Drosophila* imaginal disc development

**DOI:** 10.1371/journal.pgen.1010395

**Published:** 2022-09-27

**Authors:** Ying Feng, Yan Zhang, Zhiqing Lin, Xiaolei Ye, Xue Lin, Lixiu Lv, Yi Lin, Shenfei Sun, Yun Qi, Xinhua Lin

**Affiliations:** 1 State Key Laboratory of Optometry, Ophthalmology and Vision Science, School of Optometry and Ophthalmology and Eye Hospital, Wenzhou Medical University, Wenzhou, Zhejiang, China; 2 The Second Affiliated Hospital and Yuying Children’s Hospital of Wenzhou Medical University, Wenzhou, Zhejiang, China; 3 State Key Laboratory of Genetic Engineering, School of Life Sciences, Greater Bay Area Institute of Precision Medicine (Guangzhou), Zhongshan Hospital, Fudan University, Shanghai, China; 4 State Key Laboratory of Genetic Engineering, School of Life Sciences, Fudan University, Shanghai, China; Albert Einstein College of Medicine, UNITED STATES

## Abstract

Programmed Cell Death (PCD) or apoptosis is a highly conserved biological process and plays essential roles both in the development and stress context. In *Drosophila*, expression of pro-apoptotic genes, including *reaper (rpr)*, *head involution defective (hid)*, *grim*, and *sickle* (*skl*), is sufficient to induce cell death. Here, we demonstrate that the chromatin remodeler Dmp18, the homolog of mammalian Znhit1, plays a crucial role in regulating apoptosis in eye and wing development. We showed that loss of *Dmp18* disrupted eye and wing development, up-regulated transcription of pro-apoptotic genes, and induced apoptosis. Inhibition of apoptosis suppressed the eye defects caused by *Dmp18* deletion. Furthermore, loss of *Dmp18* disrupted H2Av incorporation into chromatin, promoted H3K4me3, but reduced H3K27me3 modifications on the TSS regions of pro-apoptotic genes. These results indicate that Dmp18 negatively regulates apoptosis by mediating H2Av incorporation and histone H3 modifications at pro-apoptotic gene loci for transcriptional regulation. Our study uncovers the role of Dmp18 in regulating apoptosis in *Drosophila* eye and wing development and provides insights into chromatin remodeling regulating apoptosis at the epigenetic levels.

## Author summary

Apoptosis is an important biological process in the development and stress context by removing unwanted or damaged cells. Dysregulation of apoptosis causes many diseases including cancers. Transcriptional induction of pro-apoptotic genes is sufficient to induce apoptosis in *Drosophila*. Znhit1 encodes a Zinc finger HIT-type containing protein and works as a component of the chromatin remodeling complex in yeast and mammals. In the current study, we identified that Dmp18, the homolog of Znhit1, regulates apoptosis by mediating the histone variant H2Av incorporation and H3K4me3 as well as H3K27me3 modifications around the transcription start site (TSS) regions of pro-apoptotic genes. *Dmp18* deletion up-regulated the transcription of pro-apoptotic genes including *rpr* and *hid*, triggered massive cell death, and resulted in eye and wing defects. Importantly, loss of *Dmp18* disrupted H2Av incorporation into chromatin, increased H3K4me3, but decreased H3K27me3 modifications on the TSS regions of pro-apoptotic genes for transcriptional activation. Together, this study reveals the mechanism by which Dmp18 regulates apoptosis in the eye and wing discs.

## Introduction

Maintaining the balance between cell death, proliferation, and differentiation is needed for organogenesis and histogenesis in multicellular organisms. Programmed Cell Death (PCD) or apoptosis plays essential roles both in the development and stress context by removing unwanted or damaged cells to keep the balance [1, 2]. Dysregulation of apoptosis induces a variety of diseases, including cancers, autoimmune diseases, and neurodegenerative diseases [3–5]. Thus, understanding the regulatory mechanisms of apoptotic process can provide insights into disease treatment and prevention [3, 6, 7]. In *Drosophila*, expression of the pro-apoptotic genes, including *reaper* (*rpr*), *head involution defective* (*hid*), *grim*, and *sickle* (*skl*), is sufficient to induce cell death [8–10], which active apoptosis by inhibiting the activity of Death-associated inhibitor of apoptosis protein 1 (DIAP1) [11–14]. Thus, understanding the underlying regulatory mechanisms of pro-apoptotic gene expression is important.

Mammals have multiple H2A variants, including H2A.Z and H2A.X. H2A.Z is considered the most universal variant and is highly conserved in eukaryotes. Deletion of *H2A*.*Z* in mice leads to embryonic death [15], suggesting an essential role of H2A.Z for embryonic development. It has been reported that H2A.Z is specifically deposited around the transcription start site (TSS) of active promoters and positively or negatively regulates gene expression [16–18]. In addition to transcriptional regulation, H2A.Z is also important for multiple chromatin-based processes including heterochromatin formation, DNA replication, nuclear reassembly, chromosome segregation, meiotic recombination initiation, and formation of higher-ordered chromosomal structures [19–28]. Recent studies have shown that H2A.Z is involved in the regulation of fear memory in mice [29–32] and tumorigenesis [33–35]. In addition to H2A.Z, H2A.X is an important component in response to DNA damage and has been shown to play important functions in biological processes such as cell division, stem cell functions, and aging [36, 37].

*Drosophila* only has a single H2A variant, H2Av, which belongs to the H2A.Z family and is necessary for animal survival [38, 39]. Its C-terminal contains an SQAY motif similar to H2A.X, suggesting that *Drosophila* H2Av may serve the functions of mammalian both H2A.Z and H2A.X in transcriptional regulation and DNA damage response [39].

The dynamic exchange of histone H2A with histone variant H2A.Z on chromatin is catalyzed by the ATP-dependent chromatin remodeling complex SWR1/SRCAP [40, 41]. Znhit1 encodes a Zinc finger HIT-type containing protein and is conserved from yeast to mammals [42, 43]. Previous studies have shown that Znhit1 works as a component of the SWR1/SRCAP complex and is involved in regulating the exchange of H2A for H2A.Z [42, 44–46]. Deletion of *Znhit1* in mice disrupts organ development and homeostasis maintenance [45–51]. However, the physiological functions of Znhit1 in other organisms remain poorly understood. Here, we used *Drosophila* as a model system and generated the *Drosophila Znhit1* (referred to as *Dmp18*) mutant fly, *Dmp18*^*d1*^, to investigate its role in eye and wing development. Our study showed that Dmp18 regulates apoptosis by controlling the transcription of pro-apoptotic genes in the eye and wing discs. We further demonstrated that Dmp18 mediates the incorporation of histone variant H2Av into chromatin and the modifications of both H3K4me3 and H3K27me3 on the TSS regions of pro-apoptotic genes for transcriptional regulation. Thus, our study reveals the role of Dmp18 in regulating apoptosis at the epigenetic levels in the eye and wing discs.

## Results

### Loss of *Dmp18* impairs eye and wing development

**Fig 1:**
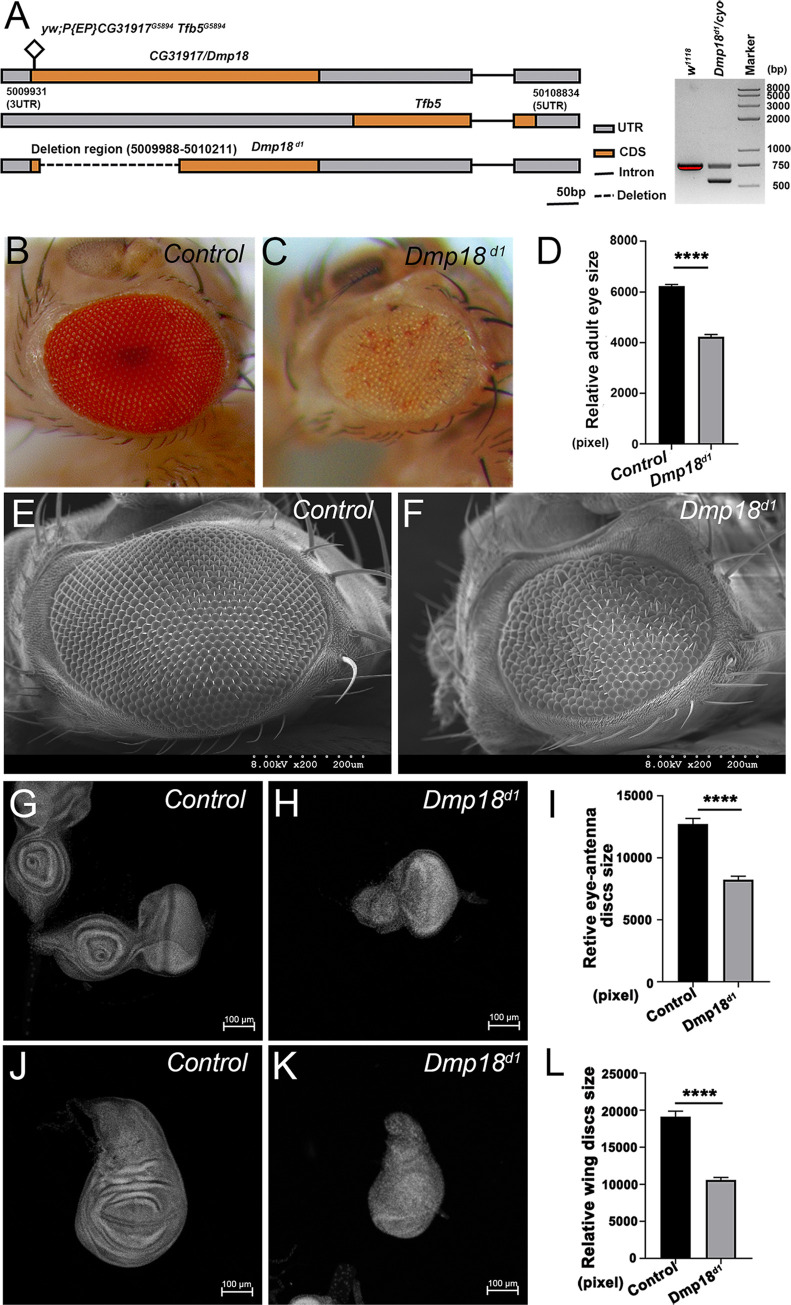
Loss of *Dmp18* disrupts eye and wing development. (A) Schematic diagram of the genomic region of *Dmp18*, *Tfb5*, and *Dmp18*^*d1*^. The square marked the P-element *yw;P{EP} CG31917*^*G5894*^ *Tfb5*^*G5894*^. The dotted line represented the knockout region which contains a 224 bp deletion. (B-F) Deletion of *Dmp18* caused eye defects. All eye phenotypes were generated by *EGUF/hid* method. (B-C) Compared with the control eye (B), loss of *Dmp18* caused small and rough eye defects (C). (D) Statistical analysis of the relative adult eye size of control (B) and *Dmp18*^*d1*^ (C). ****p<0.0001, the number of B = 30 and the number of C = 34. (E-F) Scanning electron microscope (SEM) analysis of homozygous *Dmp18*^*d1*^ eyes. (G-L) Loss of *Dmp18* caused small eye-antenna and wing discs. (G) Control eye-antenna disc. (H) Homozygous *Dmp18*^*d1*^ eye-antenna disc. (I) Statistical analysis of the relative eye-antenna disc size of control (G) and *Dmp18*^*d1*^ (H). ****p<0.0001, the number of G = 16 and the number of H = 23. (J) Control wing disc. (K) Homozygous *Dmp18*^*d1*^ wing disc. (L) Statistical analysis of the relative wing disc size of control (J) and *Dmp18*^*d1*^ (K). ****p<0.0001, the number of J = 24 and the number of K = 29. Genotypes: B and E: *yw*, *ey-Gal4*, *UAS-FLP/X; FRT*^*40A*^, *GMR-hid/FRT*^*40A*^*; UAS-CD8-GFP/+*; C and F: *yw*, *ey-Gal4*, *UAS-FLP/X; FRT*^*40A*^, *GMR-hid/FRT*^*40A*^*-Dmp18*^*d1*^*; UAS-CD8-GFP/+*; G and J: *FRT*^*40A*^*/FRT*^*40A*^; H and K: *FRT*^*40A*^*-Dmp18*^*d1*^*/FRT*^*40A*^*-Dmp18* ^*d1*^.

In our large-scaled RNA interference screening, we found that knockdown of *CG31917* caused rough and small eyes (S1B and S1C Fig). The transcript of *CG31917* encodes a protein of about 18 kD. Previous studies have shown that CG31917 is homologous with mammalian Znhit1 and was named Dmp18 in *Drosophila* [43, 52, 53]. To analyze the function of Dmp18, we generated the mutant fly (*Dmp18*^*d1*^) by P-element mediated imprecise excision, which contained a 224 bp deletion (Fig 1A). Most *Dmp18*^*d1*^ homozygotes died at the late third instar larval stage, and a few of them survived into the pupal stage. Considering that *Dmp18* is encoded in a bicistronic transcript with *Tfb5* [53], we detected the transcription of *Tfb5* by RT-qPCR in homozygous *Dmp18*^*d1*^. The result showed that the *Tfb5* transcript was still produced (S2B Fig), indicating that the expression of *Tfb5* was not interfered in homozygous *Dmp18*^*d1*^, and *Dmp18* was knocked out specifically.

To confirm the eye defects caused by *Dmp18* knockdown, we used *EGUF/hid* method [54] to generate *Dmp18* homozygous mutant adult eyes and examined the phenotype. As shown in Fig 1C and 1F, *Dmp18* deletion induced rough and small eyes as well as disorganized bristles. In addition to the impaired adult eyes, the growth rate of homozygous mutant larvae was severely delayed, and the size of eye-antenna and wing discs was reduced (Fig 1G–1L). The wing discs even disappeared in most mutant larvae. To reveal the mechanism of eye and wing defects caused by *Dmp18* deletion, we generated *Dmp18* mutant clones by *Minute* clone technique [55], which makes big mutant clones, to examine the cell differentiation in the eye disc. The differentiated photoreceptor cells express Elav in the eye disc after morphogenetic furrow (MF). Compared with wild-type cells, *Dmp18* mutant photoreceptor cells still expressed Elav (S3A–S3A” Fig), indicating that the differentiation was not affected. We further verified cell differentiation in the homozygous *Dmp18*^*d1*^ eye discs. Although some mutant discs showed an abnormal arrangement of photoreceptor cells (S3D–S3D” Fig, compared to S3B–S3B” Fig), the cell differentiation was not changed in the homozygous *Dmp18*^*d1*^ eye discs (S3C–S3C” Fig, compared to S3B–S3B” Fig).

### Loss of *Dmp18* induces apoptosis in the eye and wing discs

**Fig 2:**
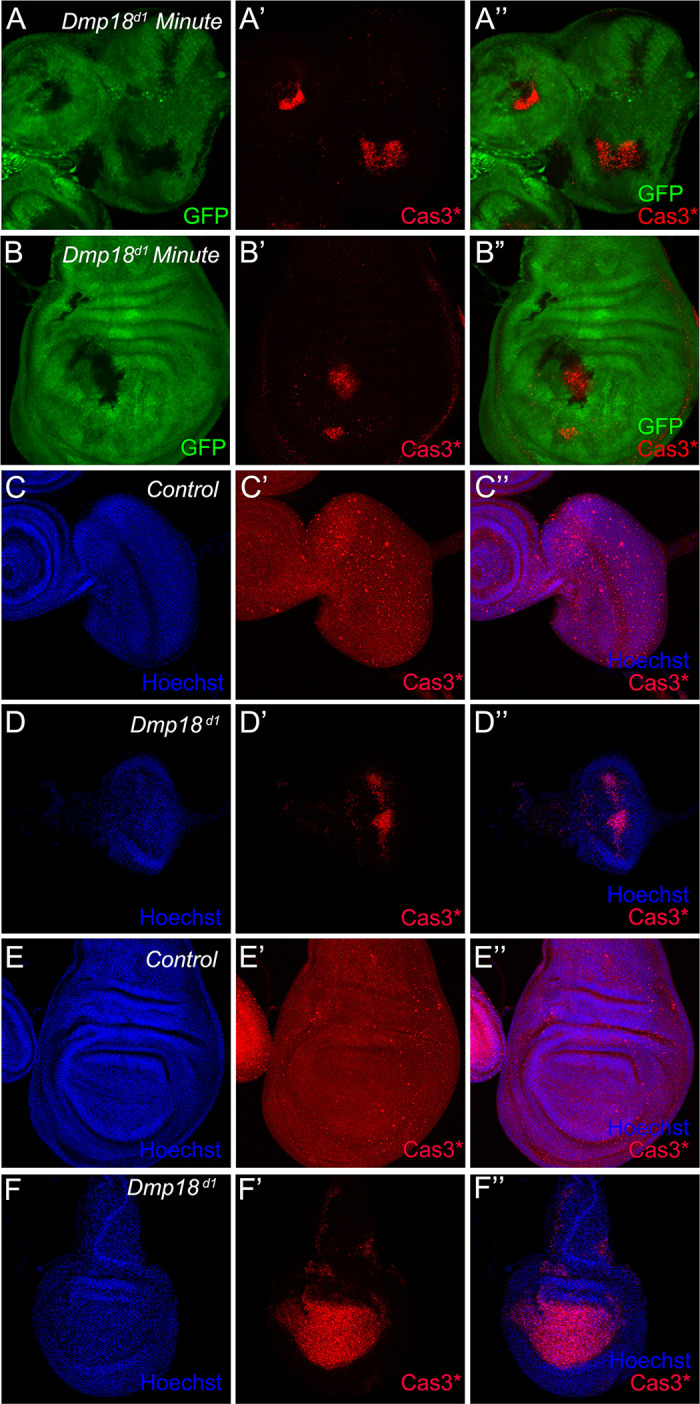
Loss of *Dmp18* induces apoptosis in the eye and wing discs. (A-B”) The Cas3* was increased in the *Dmp18* mutant clones. GFP-free region marked the *Dmp18* mutant clones. (C-D”) The Cas3* was increased in the homozygous *Dmp18*^*d1*^ eye disc (D-D”) compared to the control eye disc (C-C”). (E-F”) The Cas3* was increased in the homozygous *Dmp18*^*d1*^ wing pouch (F-F”) compared to the control wing disc (E-E”). Genotypes: A-B”: *yw*, *hs-FLP/+; FRT*^*40A*^*-M(2L)-Ubi-GFP/FRT*^*40A*^*-Dmp18*^*d1*^; C-C” and E-E”: *FRT*^*40A*^*/FRT*^*40A*^; D-D” and F-F”: *FRT*^*40A*^*-Dmp18*^*d1*^*/ FRT*^*40A*^*-Dmp18*^*d1*^.

Maintenance of the balance between cell death, differentiation, and proliferation is essential for the growth of tissues and organs. Since differentiation was not affected by *Dmp18* deletion, we speculated that the observed defects may be derived from cell death. We then examined the cell death by the cleaved Caspase 3 (Cas3*) activity in the *Dmp l8* mutant. As shown in Fig 2A–2B”, the Cas3* was increased in the *Dmp18* mutant clones both in the eye and wing discs. We also observed massive Cas3* staining in the eye-antennae disc and the wing pouch region in homozygous *Dmp18*^*d1*^ (Fig 2C–2F”). The death phenotype was also confirmed by the TdT-mediated dUTP nick end labeling (TUNEL) assay. TUNEL signals were dramatically increased after *Dmp18* deletion (S4 Fig). To verify that cell death in the eye and wing discs is indeed induced by *Dmp18* deletion, we over-expressed V5-tagged Dmp18 (*UAS-Dmp18-V5*) in the *Dmp18* mutant clones and examined the Cas3* activity. The result showed that over-expression of Dmp18-V5 completely inhibited the Cas3* activity in the *Dmp18* mutant clones (S5C–S5D”’ Fig, compared to S5A–S5B” Fig). Collectively, these data indicate that Dmp18 is required for cell survival in the eye and wing discs.

**Fig 3:**
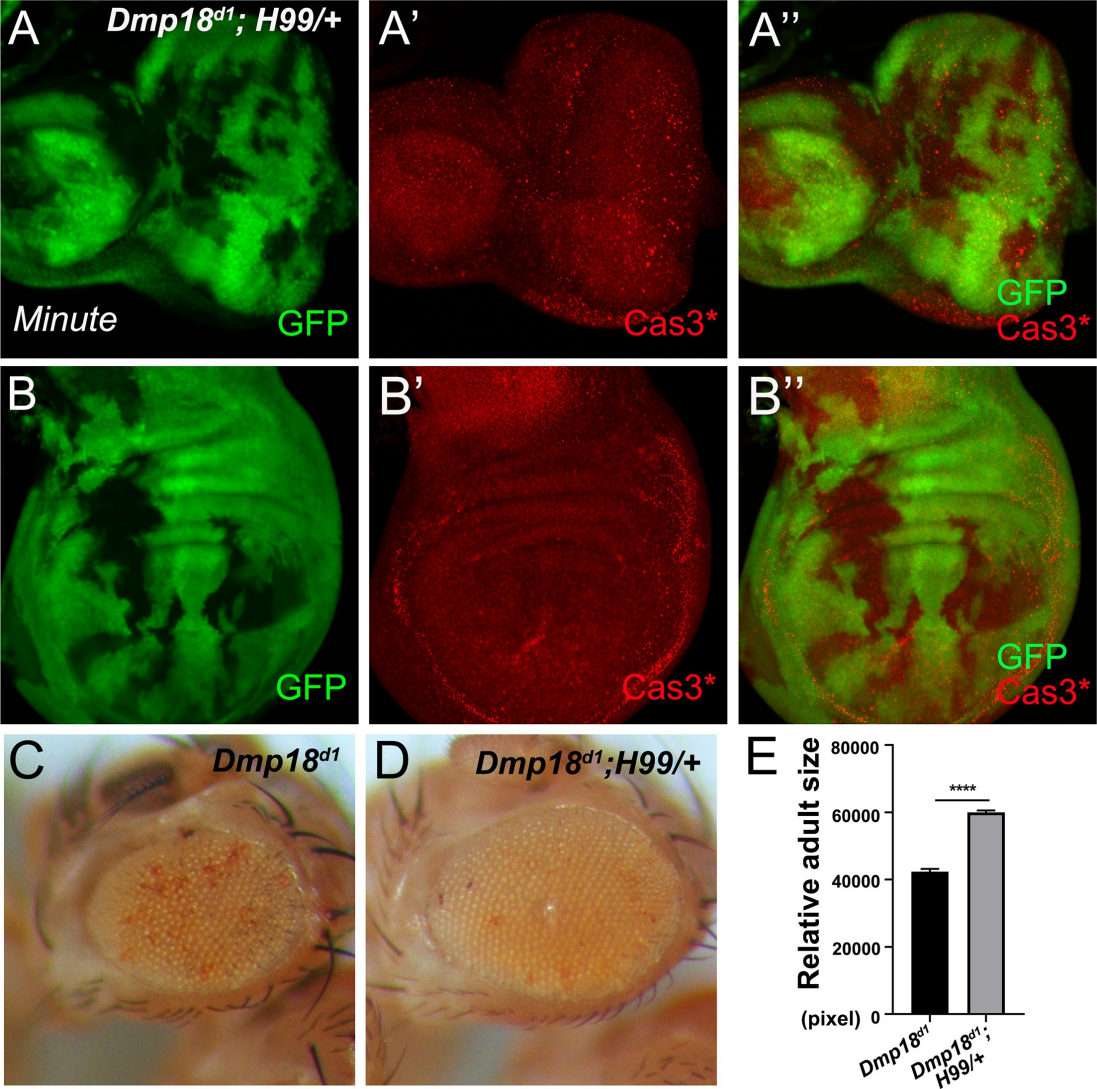
*H99* inhibits *Dmp18* deletion-induced apoptosis. (A-B”) *H99* inhibited the Cas3* activity in the *Dmp18* mutant clones both in the eye and wing discs. GFP-free region marked the *Dmp18* mutant clones. (C-D) *H99* dramatically restored the small eye defect induced by *Dmp18* deletion. Adult eye phenotypes were generated by *EGUF/hid* method. (E) Statistical analysis of the relative adult eye size of C and D. ****p<0.0001, the number of C = 34 and the number of D = 34. Genotypes: A-B”: *yw*, *hs-FLP/+; FRT*^*40A*^*-M(2L)-Ubi-GFP/FRT*^*40A*^*-Dmp18*^*d1*^*; H99/+*. C: *yw*, *ey-Gal4*, *UAS-FLP/X; FRT*^*40A*^, *GMR-hid/FRT*^*40A*^*-Dmp18*^*d1*^*; UAS-CD8-GFP/+*; D: *yw*, *ey-Gal4*, *UAS-FLP/X; FRT*^*40A*^, *GMR-hid/FRT*^*40A*^*-Dmp18*^*d1*^*; UAS-CD8-GFP/H99*.

Next, we examined whether Dmp18 controls eye and wing development by regulating apoptosis in the eye and wing discs. The deficiency line *Df(3L)H99* (referred to as *H99*) removes three pro-apoptotic genes, including *rpr*, *hid*, and *grim* (*RHG*), and suppresses apoptosis both in the development and stress response context [56]. We then investigated the function of the RHG complex in *Dmp18* deletion-induced apoptosis. The results showed that the Cas3* activity in the *Dmp18* mutant clones was significantly inhibited (Fig 3A–3B”, compared to Fig 2A–2B”) and the small eye defect caused by *Dmp18* deletion was dramatically restored by *H99* (Fig 3C–3E). Together, these results indicate that apoptosis plays a crucial role in *Dmp18* deletion-induced eye defects.

### Dmp18 regulates the transcription of pro-apoptotic genes

**Fig 4:**
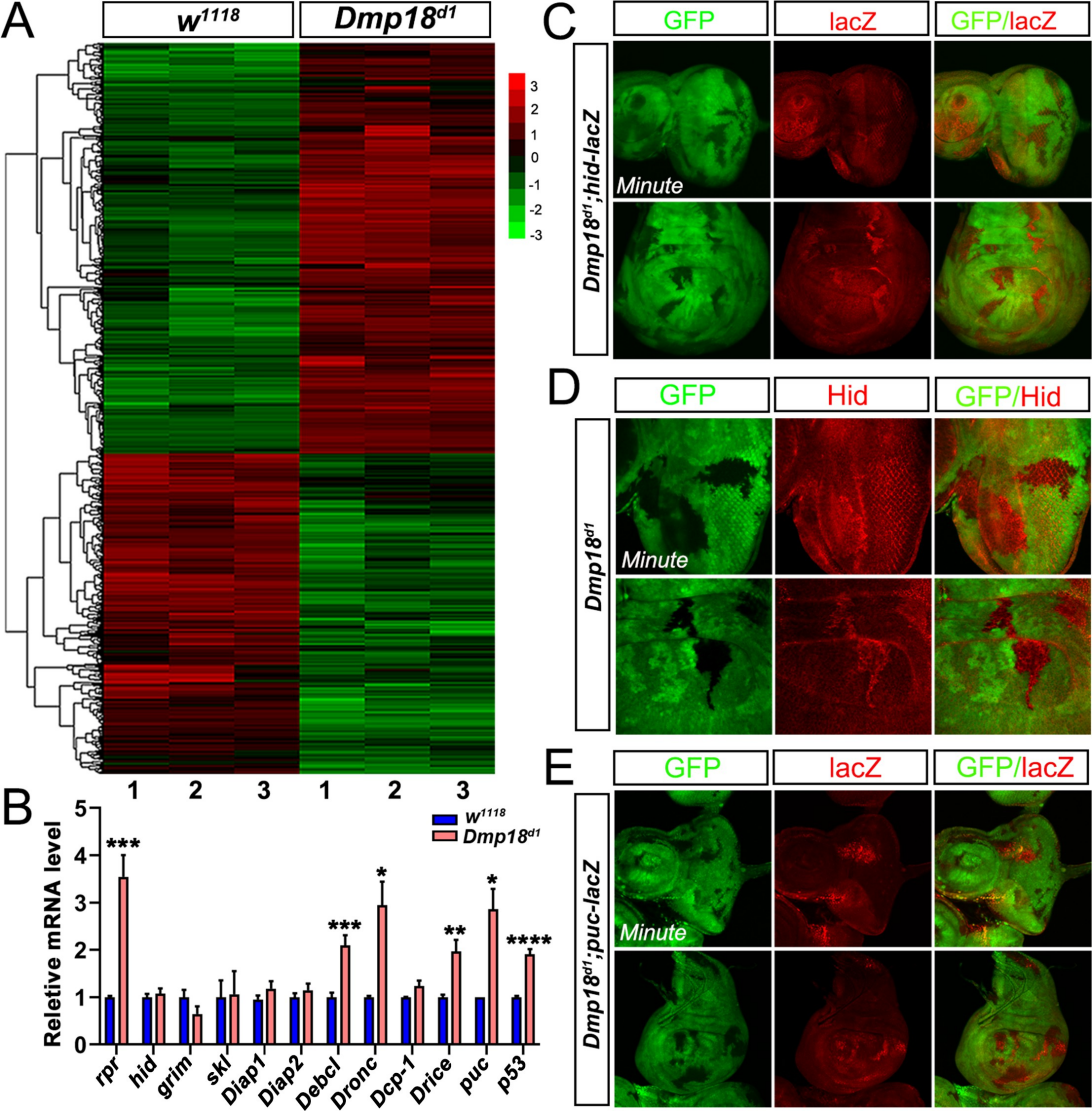
Dmp18 controls the transcription of apoptotic genes. (A) Clustered heatmap of log2-transformed FPKMs (Fragments Per Kilo bases per Million mapped fragments) showed the differentially expressed genes in homozygous *Dmp18*^*d1*^. (B) RT-qPCR analysis showed the expression changes of apoptotic-related genes in homozygous *Dmp18*^*d1*^. *p<0.05, **p <0.01, ***p<0.001 and ****p<0.0001. (C) The *hid-lacZ* was increased in the eye and wing discs in the *Dmp18* mutant clones. (D) The Hid protein was increased in the eye and wing discs in the *Dmp18* mutant clones. (E) The *puc-lacZ* was increased in the eye and wing discs in the *Dmp18* mutant clones. The GFP-free region marked the *Dmp18* mutant clones. Genotypes: C: *yw*, *hs-FLP/+; FRT*^*40A*^*-M(2L)-Ubi-GFP/FRT*^*40A*^*-Dmp18*^*d1*^*; hid-lacZ/+*; D: *yw*, *hs-FLP/+; FRT*^*40A*^*-M(2L)-Ubi-GFP/FRT*^*40A*^*-Dmp18*^*d1*^; E: *yw*, *hs-FLP/+; FRT*^*40A*^*-M(2L)-Ubi-GFP/FRT*^*40A*^*-Dmp18*^*d1*^*; puc-lacZ/+*.

Previous studies have shown that Znhit1 is involved in regulating gene transcription in organ development and homeostasis maintenance [44–46, 48–51]. To understand the mechanisms of how Dmp18 regulates apoptosis, we examined the gene expression profiles in homozygous *Dmp18*^*d1*^. The RNA-seq results showed 2253 differentially expressed genes in *Dmp18*^*d1*^, among which, 1266 genes were up-regulated and 987 genes were down-regulated (Figs 4A, S6A and S2 Table). Gene ontology (GO) and KEGG analyses performed by Metascape [57] showed that these genes are involved in multiple biological processes including tissue development, metabolic process, and stress response (S6B and S6C Fig). Of note, the expression of apoptotic-related genes, including *rpr* and *Death executioner Bcl-2* (*Debcl*), was up-regulated in homozygous *Dmp18*^*d1*^ (Fig 4A and S2 Table). The RT-qPCR results showed that, in addition to *rpr* and *Debcl*, *Death regulator Nedd2-like caspase* (*Dronc*) and the effector caspase *Death related ICE-like caspase* (*Drice*) were also up-regulated (Fig 4B). Interestingly, the transcription of inhibitor of apoptotic factors *Diap1* and *Diap2*, pro-apoptotic genes *grim* and *skl*, as well as another effector caspase Death caspase-1 (*Dcp-1*) was unchanged in *Dmp18*^*d1*^ (Fig 4B). Although, the RNA-seq and RT-qPCR analyses showed no change of *hid* transcription in whole mutant larvae (Fig 4A and 4B), the *hid-lacZ* reporter and Hid protein were increased in the *Dmp18* mutant clones (Fig 4C and 4D), indicating that Dmp18 also regulates *hid* transcription in eye and wing development. In addition, the transcription of *puc*, the target of JNK signaling, and *p53* was also up-regulated by *Dmp18* deletion (Fig 4A, 4B, 4E and S2 Table).

As both *rpr* and *hid* are up-regulated by *Dmp18* deletion (Fig 4A–4D), and *H99* inhibits *Dmp18* deletion-induced apoptosis (Fig 3A–3B”), we speculated that Dmp18 may regulate apoptosis by controlling *rpr* and *hid* transcription. Furthermore, JNK signaling and p53 can induce apoptosis by regulating transcription of *rpr* and *hid* [58–60] and were both up-regulated by *Dmp18* deletion (Fig 4A, 4B, 4E and S2 Table). Thus, we examined whether transcriptional up-regulation of *rpr* and *hid* by *Dmp18* deletion are due to the functions of JNK signaling and p53. The results showed that neither inhibition of JNK signaling by expressing dominant negative JNK (BSK^DN^) nor knocking down *p53* by RNAi could inhibit Cas3* activity in the *Dmp18* mutant clones (S7B and S8B Figs), and the expression of *hid* and *rpr* was still increased (S7D, S7E, S8C and S8D Figs). These results suggest that the up-regulated *rpr* and *hid* induced by *Dmp18* deletion were not fully due to the activation of JNK signaling or up-regulation of *p53*. Together, our data indicate that Dmp18 controls the transcription of *rpr* and *hid* to regulate apoptosis independent of JNK signaling and p53.

### Loss of *H2Av* induces apoptosis in the eye and wing discs

**Fig 5:**
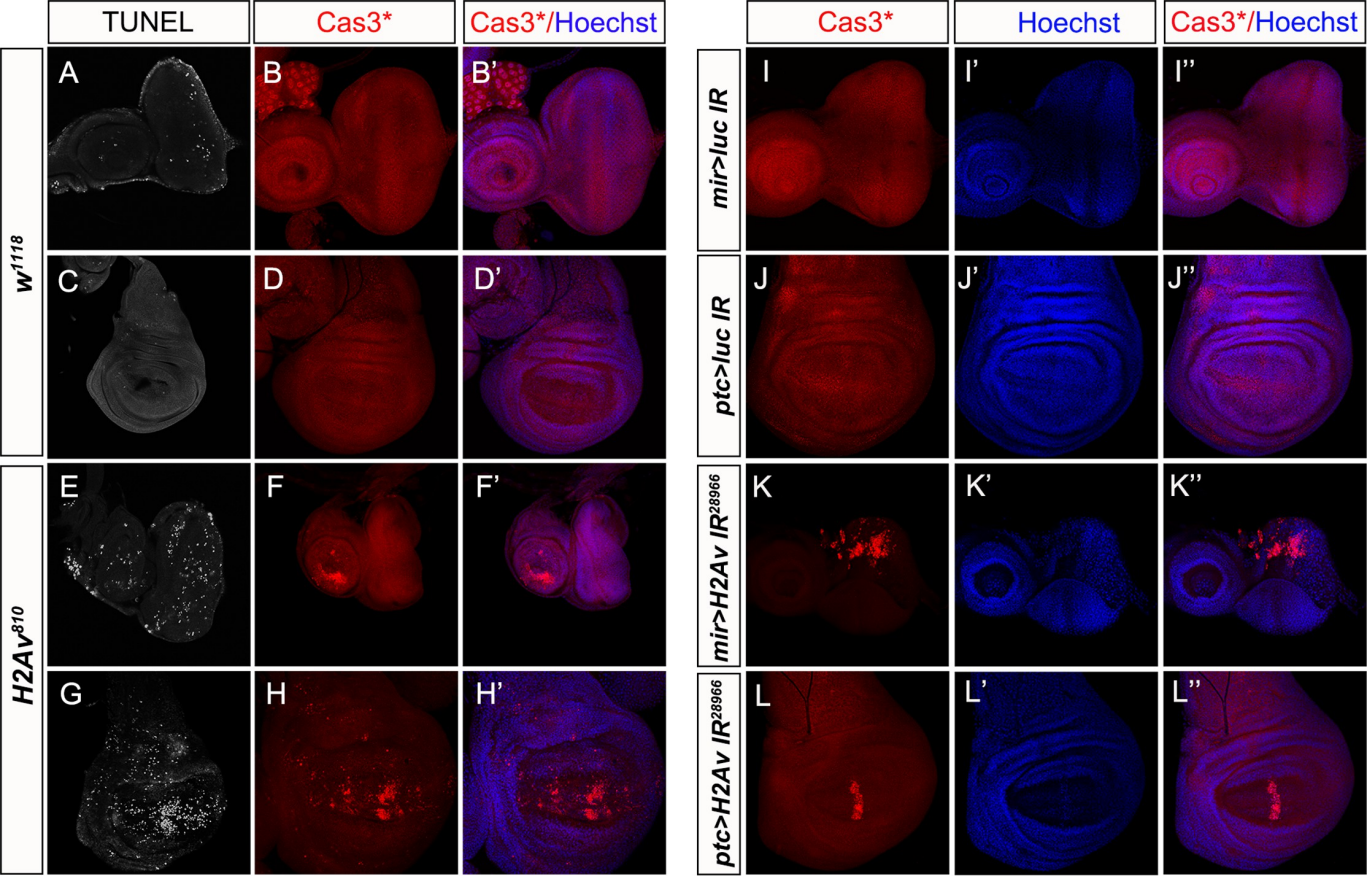
Loss of *H2Av* induces apoptosis in the eye and wing discs. (A-H’) *H2Av* deletion caused apoptosis in the eye and wing discs. (A and E) The TUNEL signals were increased in the homozygous *H2Av*^*810*^ eye disc (E) compared with the control (A). (B-B’ and F-F’) The Cas3* was activated in the antenna disc in the homozygous *H2Av*^*810*^ (F-F’) compared with the control (B-B’). (C and G) The TUNEL signals were increased in the wing pouch in the homozygous *H2Av*^*810*^ (G) compared with the control (C). (D-D’ and H-H’) The Cas3* was activated in the wing pouch in the homozygous *H2Av*^*810*^ (H) compared with the control (D). (I-L”) Knockdown of *H2Av* induced apoptosis in the eye and wing discs. (I-J”) The control eye and wing discs. (K-K”) Knockdown of *H2Av* by *mirro-gal4* induced activation of Cas3* in the dorsal compartment of the eye disc before MF. (L-L”) Knockdown of *H2Av* by *ptc-gal4* induced activation of Cas3* in the A/P boundary of the wing disc. Genotypes: A-D’: *w*^*1118*^; E-H’: *H2Av*^*810*^*/H2Av*^*810*^; I-I”: *mirror-gal4/UAS-luciferase IR*; J-J”: *ptc-gal4/+; UAS-luciferase IR/+*; K-K”: *mirror-gal4/UAS-H2Av IR*^*28966*^; L-L”: *ptc-gal4/+; UAS-H2Av IR*^*28966*^*/+*.

**Fig 6:**
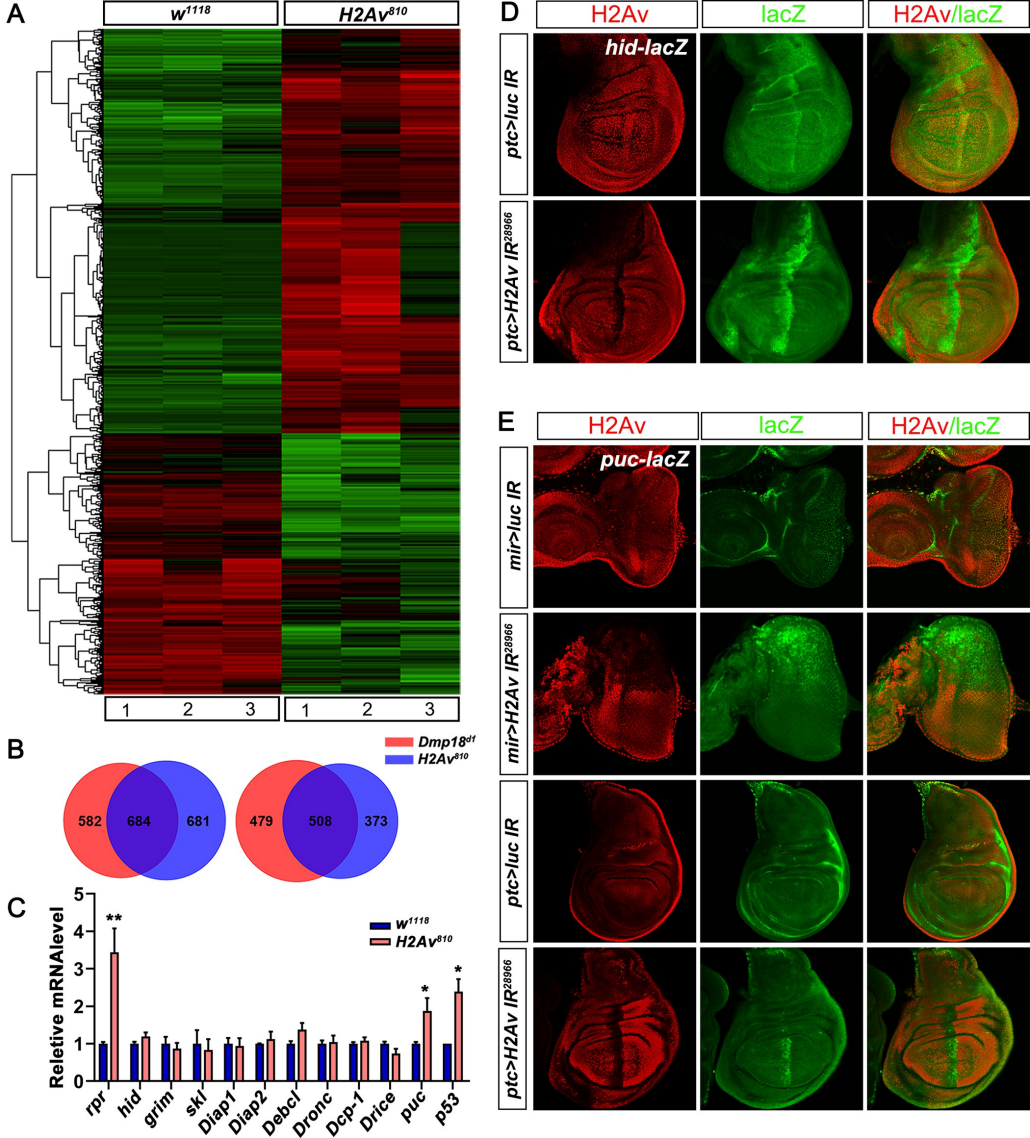
H2Av regulates the transcription of apoptotic genes. (A) Clustered heatmap of log2-transformed FPKMs showed the differentially expressed genes in homozygous *H2Av*^*810*^. (B) Venn diagram showed the overlap of differentially expressed genes in *Dmp18*^*d1*^ and *H2Av*^*810*^. (C) RT-qPCR analysis showed the expression changes of apoptotic-related genes in homozygous *H2Av*^*810*^. *p<0.05 and **p<0.01. (D) Knockdown of *H2Av* by *ptc-gal4* increased *hid-lacZ* expression. (E) Knockdown of *H2Av* by *mirr-gal4* or *ptc-gal4* increased *puc-lacZ* expression. Genotypes: D: *ptc-gal4/+; UAS-luciferase IR/hid-lacZ* and *ptc-gal4/+; UAS-H2Av IR*^*28966*^*/hid-lacZ*. E: *mirror-gal4*, *puc-lacZ/UAS-luciferase IR*, *mirror-gal4*, *puc-lacZ/UAS-H2Av IR*^*28966*^, *ptc-gal4/+; UAS-luciferase IR/puc-lacZ* and *ptc-gal4/+; UAS-H2Av IR*^*28966*^*/puc-lacZ*.

As a subunit of the SRCAP chromatin remodeling complex, Znhit1 has been reported to regulate H2A.Z incorporation into chromatin, thus controlling gene expression [42, 44–46, 48, 50, 51]. H2Av is the only H2A variant in *Drosophila* [39]. To investigate whether apoptosis induced by *Dmp18* deletion is related to H2Av, we examined the function of H2Av in regulating apoptosis. Same as *Dmp18* deletion, loss of *H2Av* (*H2Av*^*810*^) [38] induced animal death at the late third instar larval stage and resulted in developmental delay. Knockout or knockdown of *H2Av* increased the Cas3* activity and TUNEL signals both in the eye and wing discs (Fig 5). The RNA-seq results revealed that *H2Av* deletion induced 2246 differentially expressed genes, among which, 1365 genes were up-regulated and 881 genes were down-regulated (Figs 6A, S9A and S3 Table). The GO and KEGG analyses [57] showed that the H2Av-regulated genes are involved in the regulation of multiple biological processes such as tissue development, metabolic process, and stress response (S9B and S9C Fig), which is similar to the Dmp18-regulated genes. Compared with the 2253 Dmp18-regulated genes from RNA-seq, they shared 684 up-regulated genes and 508 down-regulated genes (Fig 6B). The transcription of *rpr*, *hid*, *puc* and *p53* was up-regulated in homozygous *H2Av*^*810*^ (Fig 6A, 6C, and S3 Table). Interestingly, the *Debcl*, *Dronc*, and *Drice* were highly expressed in *Dmp18* deletion, but were not changed in *H2Av* deletion (Fig 6C). The *hid-lacZ* and *puc-lacZ* were also increased when *H2Av* was knocked down by *mirro-gal4* or *ptc-gal4* in the eye and wing discs (Fig 6D and 6E). Moreover, the *H99* inhibited the apoptosis induced by *H2Av* knockdown (S11 Fig). Inhibition of JNK signaling or knockdown of *p53* failed to suppress apoptosis induced by *H2Av* knockdown, and the expression of *hid-lacZ* was still up-regulated (S12 and S13 Figs). These data indicate that *H2Av* deletion largely resembles the phenotype of *Dmp18* deletion, and it regulates apoptosis by controlling the expression of pro-apoptotic genes.

### Dmp18 regulates apoptosis by mediating H2Av incorporation

**Fig 7:**
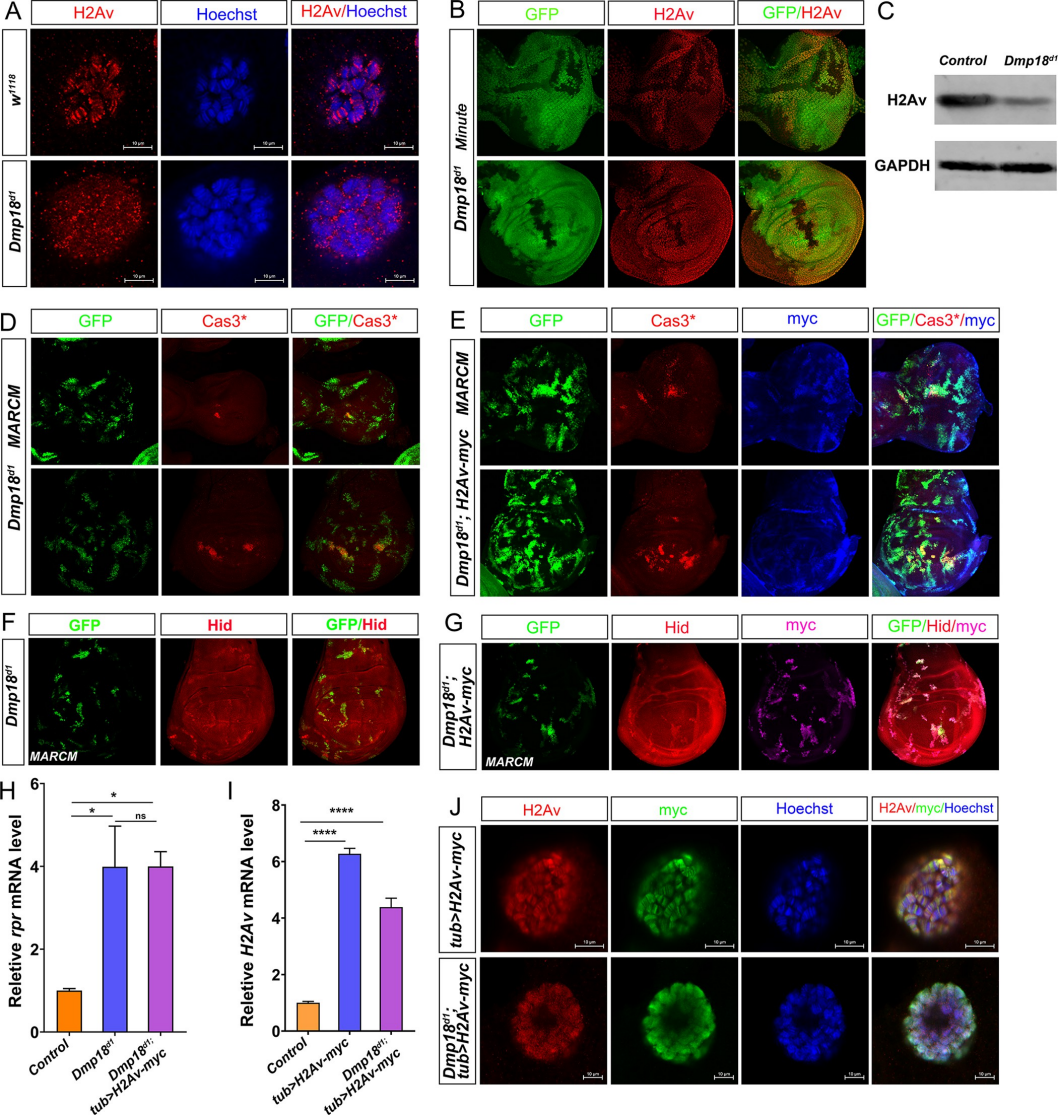
Dmp18 regulates apoptosis by mediating H2Av incorporation. (A) Loss of *Dmp18* disrupted incorporation of H2Av into chromatin. (B) The protein level of H2Av was reduced in the *Dmp18* mutant clones in the eye and wing discs. GFP-free region marked the *Dmp18* mutant clones. (C) The whole larvae were used for immunoblotting with the H2Av antibody. The protein level of H2Av was reduced in the homozygous *Dmp18*^*d1*^. The GAPDH worked as the loading control. (D) Loss of *Dmp18* activated Cas3* in the eye and wing discs. (E) Over-expression of H2Av-myc did not inhibit Cas3* activity in the *Dmp18* mutant clones. (F) Loss of *Dmp18* up-regulated Hid expression. (G) Over-expression of H2Av-myc did not reduce the Hid expression in the *Dmp18* mutant clones. D-G: GFP marked the *Dmp18* mutant clones or *Dmp18* mutant clones with over-expressed H2Av-myc. (H) The RT-qPCR analysis showed the transcription level of *rpr* in the control, homozygous *Dmp18*^*d1*^ and homozygous *Dmp18*^*d1*^ with over-expressed H2Av-myc. The *rpr* still showed high expression when H2Av-myc was over-expressed in the homozygous *Dmp18*^*d1*^. (I) The RT-qPCR analysis showed the transcription of *H2Av* in control, H2Av-myc and homozygous *Dmp18*^*d1*^ with over-expressed H2Av-myc. (J) H2Av-myc driven by *tub-gal4* was deposited on chromatin and co-localized with endogenous H2Av well in the wild-type, but it failed to be deposited on chromatin in the homozygous *Dmp18*^*d1*^. *p<0.05 and ****p<0.0001. Genotypes: A: *w*^*1118*^ and *FRT*^*40A*^-*Dmp18*^*d1*^*/FRT*^*40A*^-*Dmp18*^*d1*^; B: *yw*,*hs-FLP/+; FRT*^*40A*^*-M(2L)-Ubi-GFP/FRT*^*40A*^*-Dmp18*^*d1*^; D and F: *yw*, *hs-FLP*, *tub-Gal4*, *UAS-nls-GFP/+; tub-Gal80*, *neoFRT*^*40A*^*/FRT*^*40A*^*-Dmp18*^*d1*^; E and G: *yw*, *hs-FLP*, *tub-Gal4*, *UAS-nls-GFP/+; tub-Gal80*, *neoFRT*^*40A*^*/FRT*^*40A*^*-Dmp18*^*d1*^*; UAS-H2Av-myc/+*; J: *tub-gal4/UAS-H2Av-myc* and *FRT*^*40A*^-*Dmp18*^*d1*^*/FRT*^*40A*^-*Dmp18*^*d1*^*; tub-gal4/UAS-H2Av-myc*.

Since *Dmp18* deletion and *H2Av* deletion generate similar phenotype, we hypothesized that Dmp18 regulates apoptosis by controlling H2Av deposition. To test our hypothesis, we examined the H2Av deposition on the polytene chromosome in the homozygous *Dmp18*^*d1*^. As expected, loss of *Dmp18* disrupted H2Av deposition on chromatin (Fig 7A). Surprisingly, the protein level of H2Av was dramatically reduced in the *Dmp18* mutant clones in the eye and wing discs (Fig 7B) and whole mutant larvae (Fig 7C), suggesting that Dmp18 not only controls H2Av deposition on chromatin but also regulates its protein level. To rule out the possibility that the apoptosis triggered by *Dmp18* deletion is due to the degradation of H2Av, we generated the transgenic fly (*UAS-H2Av-myc*) and performed the rescue experiment with an over-expression of H2Av-myc in the *Dmp18* mutant clones to examine the Cas3* activity. The result showed that over-expression of H2Av-myc alone did not activate Cas3* (S14B–S14B”’ Fig), and over-expression of H2Av-myc in the *Dmp18* mutant clones failed to inhibit Cas3* activity (Fig 7E, compared to Fig 7D). The expression of Hid and *rpr* still increased when H2Av-myc was over-expressed in the *Dmp18* mutant clones (Fig 7G) or homozygous *Dmp18*^*d1*^ (Fig 7H). We further examined the deposition of H2Av-myc on the polytene chromosome in the wild-type and homozygous *Dmp18*^*d1*^. H2Av-myc driven by *tub-gal4* was incorporated into chromatin and co-localized with endogenous H2Av well in the wild-type, but it could not be deposited on chromatin in the homozygous *Dmp18*^*d1*^ (Fig 7J). Together, these data argue that Dmp18 regulates apoptosis by mediating H2Av incorporation.

**Fig 8:**
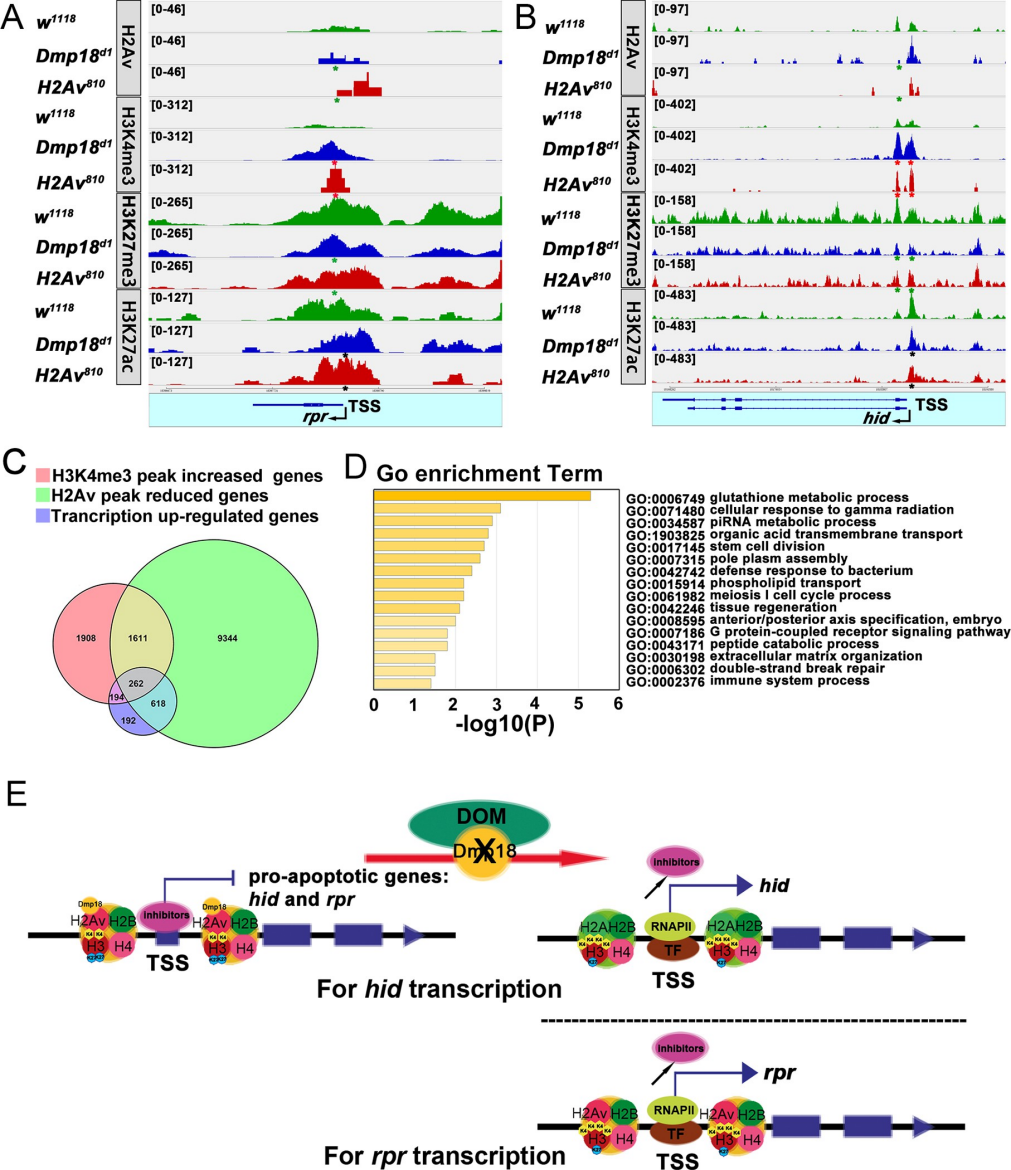
Dmp18 mediates H2Av incorporation and histone H3 modifications at the TSS regions of pro-apoptotic genes for transcriptional regulation. (A and B) The CUT&Tag assay and sequencing were performed to examine the binding of H2Av (row 1 to 3), H3K4me3 (row 4 to 6), H3K27me3 (row 7 to 9), and H3K27ac (row 10 to 12) at the TSS regions of *rpr* and *hid* in *w*^*1118*^, homozygous *Dmp18*^*d1*^ and homozygous *H2Av*^*810*^. The binding of H2Av at the TSS region of *rpr* (A) was not changed, but its binding at *hid* (B) was reduced in *Dmp18*^*d1*^ and *H2Av*^*810*^ (green asterisks marked). The binding of H3K4me3 at the TSS regions of *hid* and *rpr* was increased in *Dmp18*^*d1*^ and *H2Av*^*810*^ (red asterisks marked). The binding of H3K27me3 at the TSS regions of *hid* and *rpr* was slightly reduced in *Dmp18*^*d1*^ and *H2Av*^*810*^ (green asterisks marked). The binding of H3K27ac at the TSS regions of *rpr* and *hid* was not obviously changed (black asterisks marked). (C) Venn diagram showed the overlap of H2Av-binding reduced genes, H3K4me3-binding increased genes, and Dmp18 up-regulated genes. (D) GO analysis of the overlapped genes in C. (E) The working model of Dmp18 regulating apoptosis in *Drosophila*: Dmp18 regulates apoptosis by mediating H2Av incorporation and histone H3 modifications at the TSS regions of pro-apoptotic genes for transcriptional regulation. For *hid* transcription, Dmp18 mediates the H2Av incorporation and both H3K4me3 and H3K27me3 modifications for transcriptional regulation. For *rpr* transcription, Dmp18 directly or indirectly mediates H3K3me4 and H3K27me3 modifications for transcriptional regulation.

Previously, it has been shown that H2Av is particularly enriched downstream of TSS in *Drosophila* genes to regulate transcription [61]. To determine whether Dmp18 regulates pro-apoptotic gene transcription by controlling H2Av incorporation into the genome, we isolated the eye and wing discs from *w*^*1118*^, *Dmp18*^*d1*^, and *H2Av*^*810*^, and performed the Cleavage Under Targets and Tagmentation (CUT&Tag) assay and sequencing. The result showed that the H2Av-binding peaks around the TSS of *rpr* were lower than that of *hid* in wild-type (Fig 8A and 8B, row 1). The binding of H2Av at downstream of *hid* TSS region was almost lost in the homozygous *Dmp18*^*d1*^ and *H2Av*^*810*^ (Fig 8B, row 2 and 3), but its binding around the TSS of *rpr* was not obviously changed (Fig 8A, row 2 and 3). These data indicate that Dmp18 regulates H2Av deposition on the TSS region of *hid*, but not *rpr*.

### Dmp18/H2Av regulates histone modifications at the TSS of pro-apoptotic genes

To explore how Dmp18/H2Av affects pro-apoptotic gene transcription, we examined the epigenetic modifications of H3 at *rpr* and *hid* loci by the CUT&Tag assay and sequencing in *w*^*1118*^, *Dmp18*^*d1*^, and *H2Av*^*810*^. Considering the transcriptional up-regulation of *rpr* and *hid* in these mutants, we first intended to examine the binding of active histone marks H3K4me3 and H3K27ac at *rpr* and *hid* loci. As shown in Fig 8A and 8B, the binding of H3K4me3 at the TSS regions of *rpr* and *hid* was significantly increased in homozygous *Dmp18*^*d1*^ and *H2Av*^*810*^, but the binding of H3K27ac was not obviously changed (Fig 8A and 8B). We further examined the binding of silencing histone mark H3K27me3 at the TSS regions of *rpr* and *hid*. The result revealed that the binding of H3K27me3 at the TSS region of *hid* and *rpr* was slightly reduced in homozygous *Dmp18*^*d1*^ and *H2Av*^*810*^ (Fig 8A and 8B). These results indicate that Dmp18/H2Av regulates *rpr* and *hid* transcription by mediating the H3K4me3 and H3K27me3 modifications at the TSS regions.

## Discussion

Znhit1 is a component of the SRCAP chromatin remodeling complex and is involved in catalyzing the exchange of H2A with H2A.Z in chromatin, thus regulating gene transcription [42, 44–46, 48, 50, 51]. Dmp18, the *Drosophila* Zinc finger HIT-type containing protein, is the homolog of mammalian Znhit1 [43, 52, 53]. In the current study, we used *Drosophila* as a model system to investigate its function in imaginal disc development. Our study revealed a model for Dmp18 regulating apoptosis in the eye and wing discs: Dmp18 regulates the transcription of pro-apoptotic genes, *hid* and *rpr*, to control the apoptotic process. For *hid* transcription, *Dmp18* deletion disrupts H2Av incorporation into downstream of the TSS region, increases H3K4me3, but reduces H3K27me3 modifications to induce transcriptional up-regulation. For *rpr* transcription, *Dmp18* deletion directly or indirectly increases H3K4me3 but reduces H3K27me3 modifications at the TSS region to induce transcriptional up-regulation (Fig 8E).

### Dmp18/H2Av regulates apoptosis in the specific regions of the eye and wing discs

Our data in this study uncover the function of Dmp18 regulating apoptotic process by controlling the transcription of pro-apoptotic genes in the eye and wing discs. *Dmp18* deletion induced eye and wing defects and triggered massive cell death (Figs 1B–1L, 2 and S4). We further verified that the histone variant, H2Av, is also involved in regulating cell death in the eye and wing discs (Fig 5). Interestingly, cell death induced by *Dmp18* or *H2Av* deletion specifically occurred in the wing pouch and before MF in the eye disc (Figs 2, 5 and S4), indicating that Dmp18 and H2Av regulate apoptosis in a cell-type-specific manner. This data is consistent with the previous report that the wing pouch cells are more sensitive to apoptosis [62]. One possibility is that the different cells may express different protective factors in response to apoptosis, and the wing pouch cells may have some particular apoptotic regulators [63]. In the eye disc, the differentiation was not affected after *Dmp18* deletion (S3 Fig), and cell death was not detected in the photoreceptor cells after *Dmp18* or *H2Av* deletion (Figs 2 and 5), indicating that Dmp18 and H2Av may regulate cell death in rapidly growth cells, but not in differentiated cells.

Although phenotypic similarity is shared by *Dmp18*^*d1*^ and *H2Av*^*810*^, *Dmp18*^*d1*^ shows more severe defects in the eye and wing discs. Compared to the small eye and almost disappeared wing discs in homozygous *Dmp18*^*d1*^ (Fig 1G–1L), the size of eye and wing discs in the *H2Av*^*810*^ was not reduced. *Dmp18*^*d1*^ induces massive cell death before MF in the eye disc and the wing pouch (Figs 2 and S4). However, *H2Av*^*810*^ shows moderate cell death in the wing pouch and antenna region (Fig 5A–5H’). *H2Av*^*810*^ mutant bears a 311 bp deletion including the second exon. The deletion does not alter the reading frame, and a truncated protein could still be produced, which is 26 amino acids shorter than the wild-type H2Av [38]. The H2Av expression could still be detected in *H2Av*^*810*^ in some salivary gland cells by the commercial antibody (S15C–S15D’ Fig). This might count for a less severe phenotype in homozygous *H2Av*^*810*^ compared with that of homozygous *Dmp18*^*d1*^.

### Dmp18/H2Av regulates apoptosis by controlling the transcription of pro-apoptotic genes

Transcriptional up-regulation of pro-apoptotic genes is sufficient to induce apoptosis. Our current work suggests that Dmp18 and H2Av regulate apoptosis by controlling pro-apoptotic gene expression. The RNA-seq and RT-qPCR analyses showed that the transcription of *rpr* was significantly increased both in *Dmp18*^*d1*^ (Fig 4A, 4B and S2 Table) and *H2Av*^*810*^ (Fig 6A, 6C and S3 Table). The changes of *hid* expression could be detected in the *Dmp18* mutant clones (Fig 4C and 4D), but was not detected in the whole mutant larvae by RNA-seq and RT-qPCR (Fig 4A, 4B and S2 Table). The RT-qPCR results from a single larva showed that the transcription of *hid* showed different changes in homozygous *Dmp18*^*d1*^ compared with different control groups (S16 Fig), indicating that the transcription of *hid* may have individual differences. Another possibility is that the expression of *hid* may be only up-regulated in imaginal discs after *Dmp18* deletion. It has been reported that *hid* is regulated by distinct mechanisms in different tissues [64, 65]. Due to the small eye disc and almost lost wing disc in homozygous *Dmp18*^*d1*^, the change of *hid* expression is hard to be detected in whole mutant larvae. Most interestingly, the up-regulated *hid* by *Dmp18* deletion in the eye and wing discs was not confined to the wing pouch and before MF in the eye-antenna disc (Fig 4C and 4D), but the Cas3* is only activated in these regions, indicating that the *hid* expression in the wing and eye discs is not entirely overlapped with regions of cell death. One possibility is that different cells may have different sensitivity to the amount of *hid* expression to induce apoptosis in the eye and wing discs.

Multiple signals and factors are involved in regulating the transcription of pro-apoptotic genes such as JNK signaling and p53, which regulate apoptosis by controlling the transcription of *rpr* and *hid* [58–60]. Loss of *Dmp18* or *H2Av* activated the JNK signaling and up-regulated *p53* expression (Figs 4A, 4B, 4E, 6A, 6C and 6E). However, inhibition of JNK signaling or knockdown of *p53* failed to suppress cell death, and the expression of *rpr* or *hid* was still up-regulated in the mutants (S7, S8, S12 and S13 Figs). These results suggest that Dmp18 and H2Av regulate the transcription of pro-apoptotic genes independent of JNK signaling and p53.

### Dmp18/H2Av regulates gene transcription by mediating the histone modifications

Znhit1 regulates gene transcription by controlling the incorporation of H2A.Z [42, 44–46, 48, 50, 51]. Our current study showed that loss of *Dmp18* disrupted H2Av incorporation into chromatin (Fig 7A). The CUT&Tag assay showed that the binding of H2Av at downstream of *hid* TSS region was lost in *Dmp18*^*d1*^ and *H2Av*^*810*^ (Fig 8B), but its binding at the TSS region of *rpr* appeared to no change (Fig 8A). These data suggest that Dmp18 regulates *hid* transcription by incorporating H2Av into TSS, but controls the transcription of *rpr* by different mechanism(s). The results from the anti-H2Av CUT&Tag assay showed that the H2Av-binding peaks on the *rpr* locus were lower than those of *hid* in wild-type, indicating that *rpr* may belong to the low H2Av-binding gene relative to *hid*. Our data revealed that loss of *Dmp18* has limited effects on H2Av incorporation in low H2Av-binding genes such as *rpr*. Although the H2Av binding at *rpr* locus is not changed, the active histone mark H3K4me3 is increased and the silencing histone mark H3K27me3 is slightly reduced at the TSS region of *rpr*, which may induce the transcriptional up-regulation of *rpr*. These results suggest that Dmp18 may control *rpr* transcription via being involved in regulating histone modifications. Further studies are needed to define the involved mechanisms.

In *Drosophila*, the single DOMINO chromatin remodeling complex combines the functions of histone acetylation activity and histone variant exchange [66, 67]. H2Av incorporates into downstream of TSS by DOMINO, and the amount of H2Av correlates with the open chromatin structure and gene expression [61, 68]. H2Av incorporation into promoters can reduce the nucleosome barrier to RNAPII and promote transcription elongation [68]. Loss of *H2Av* or *domino* causes a global down-regulation of gene expression [67, 69], especially down-regulation of housekeeping gene transcription during Zygotic Genome Activation (ZGA) [69]. However, a few reports have shown that H2Av is involved in gene repression. *H2Av* deletion enhanced the phenotypes of *Polycomb* (*Pc*) mutation and suppressed of the phenotypes of *Trithorax* mutant [19]. H2Av cell-specifically binds to the enhancer elements and represses *Distalless* (*Dll*) transcription in *Drosophila* embryo [70]. Here, we demonstrated that *Dmp18* or *H2Av* deletion leads to high expression of pro-apoptotic genes by modifying the binding of epigenetic marks H3K4me3 and H3K27me3 at gene loci (Fig 8A and 8B), which provides a new insight into how Dmp18/H2Av regulates gene transcription.

## Materials and methods

### *Drosophila* strains and genetics

All *Drosophila* strains were kept and crossed at 25°C. The *Dmp18* mutant fly (*Dmp18*^*d1*^) was generated from *yw;P{EP}CG31917*^*G5894*^ *Tfb5*^*G5894*^ (BL26915) by P-element-mediated imprecise excision which deleted 224 bp. The primer used to detect the deletion as followed: *Forward*: *5’-CTATCGATGACGCTCACAGC-3’*; *Reverse*: *5’-CGACAAGGACGCTTAGAACC-3’*. The fly lines: *hid-lacZ*, *puc-lacZ*, *mirror-Gal4*, *ptc-Gal4*, *en-Gal4*, *tubulin-Gal4*, *H2Av*^*810*^ (BL9264), *UAS-H2Av IR* (BL28966 and BL44056), *UAS-luciferase IR*, *Df(3L)H99*, *UAS-BSK*^*DN*^ (BL6409 and BL9311), *yw*, *hs-FLP; FRT*^*40A*^*-M(2L)-Ubi-GFP/BCG* and *yw*, *ey-Gal4*, *UAS-FLP; neoFRT*^*40A*^, *GMR-hid/cyo;UAS-CD8-GFP/TM2* were obtained from Bloomington Drosophila Stock Center (Department of Biology, Indiana University, Bloomington, IN, USA). The *H2Av*^*810*^ was described previously [38]. The *Df(3L)H99* is a deficiency line that deleted the pro-apoptotic genes including *rpr*, *hid*, and *grim* [56]. The *yw*,*hs-FLP*,*tub-Gal4*,*UAS-nlsGFP/FM7;tub-Gal80*,*neoFRT*^*40A*^*/cyo* (for MARCM analysis, TB00132) and *UAS-p53 IR* (THU2533 and THU5318) were obtained from Tsing Hua Fly Center (Tsing Hua University, Beijing, China). The *UAS-Dmp18-V5* and *UAS-H2Av-myc* transgenic flies were generated in this study.

### Minute Clone and MARCM Clone

The *Dmp18* mutant clones were generated by the FLP/FRT method [71]. For Minute clone and MARCM clone experiments, the flies were cultured at 25°C and heat-shocked at 37°C in water bath for 60 min at 24 h and 48 h after crossed. The flies were then cultured at 25°C to third instar larvae.

### EGUF/hid

EGUF/hid method was described as previous [54]. Briefly, the mutant flies *FRT*^*40A*^*-Dmp18*^*d1*^*/cyo* crossed with tool line *yw*, *ey-Gal4*, *UAS-FLP; neoFRT*^*40A*^, *GMR-hid/cyo; UAS-CD8-GFP/TM2*. The crosses were maintained at 25°C to adult fly and examined the eye phenotype.

### Immunostaining

The eye and wing imaginal discs were dissected from the later third instar larvae stage, fixed, and stained as standard procedure. The following antibodies were used: rabbit anti-cleaved Caspase-3 (1:200, Cell Signaling Technology, 9661), mouse anti-V5 (1:500, Invitrogen, R960-25), chicken anti-beta Galactosidase (1:1000, Abcam, ab9361), rabbit anti-Hid (d-300) (1:20, Santa Cruz Biotechnology, sc-33744), rabbit anti-H2Av (1:200, Active Motif, 39715), goat anti-Myc (1:200, Abcam, ab9132), rat anti-Elav (1:200, DSHB, 7E8A10). The primary antibodies were detected by fluorescent-conjugated secondary antibodies from Invitrogen (Waltham, MA, USA). The nuclear was detected by Hoechst 33258 (Sigma, St. Louis, MO, USA). The TUNEL assay kit was from Roche (Indianapolis, IN, USA) and performed as described previously [72]. All pictures were taken by confocal laser scanning microscope (LSM710 and LSM880, Carl Zeiss, Jena, Germany).

### The eye imaginal disc, wing imaginal disc and adult eye size quantification and statistical analysis

All animals were crossed at the same time and raised under identical circumstances. The eye and wing imaginal discs were obtained from the third instar larvae by randomly with the correct genotype, and the pictures were taken by confocal laser scanning microscope at the same magnification. The adult eyes were obtained from females by microscope (SteREO Discovery V12, Carl Zeiss) at the same magnification. For SEM, adult flies were anesthetized, mounted on the stage, and observed under the scanning electron microscope (VE-7800, keyence, Tokyo, Japan) in the vacuum mode. The pictures were taken at the same magnification. The relative size of eye discs, wing discs, and adult eyes was measured by Photoshop software (Adobe Systems Inc., San Jose, CA, USA). For size measure, we used the wand tool in Photoshop to select the region that needs to be measured and recorded the pixel value in the histogram (cache level is set to 1). The pixel value represented the relative size. All data were analyzed by Graphpad Prism 8 (San Diego, CA, USA), using the two-tailed unpaired t-test or the Mann Whitney test (means ± SEM). *p*<0.05 was considered statistically significant.

### Quantitative RT-PCR (RT-qPCR)

Total RNA was extracted from 1 third instar larva (for single larva qPCR) or 3–5 third instar larvae with TRIzol (Invitrogen, Carlsbad, CA, USA). The complementary DNA (cDNA) was prepared with the M-MLV Reverse transcriptase (Applied Biosystems, Foster City, CA, USA). The qPCR reactions were performed using SYBR select Master Mix (Applied Biosystems, Foster City, CA, USA) on Quant Studio 5 (Applied Biosystems, Foster City, CA, USA). Quantification was normalized to *RPL32*. The data represented an average of at least three independent assays and were analyzed by Graphpad Prism 8 (San Diego, CA, USA), using the two-tailed unpaired t-test or Mann Whitney test for two groups and one-way ANOVA with Tukey’s multiple comparisons test for multiple groups (means ± SEM). *p*<0.05 was considered statistically significant. The primers used for qPCR were listed in the S1 Table.

### RNA-Seq

RNA from 10 third instar larvae were isolated by TRIzol and then converted into cDNA libraries. High-throughput sequencing was performed using Illumina Novaseq 6000 platform for 3 biological replicates. Over 40 million reads were obtained for each sample. The RNA-seq data were mapped to *Drosophila* BDGP6 version 92 reference genome by HISAT2, the uniquely mapped reads were used to estimate the expression values at the gene level by FPKMs. Statistical significant tests of differentially expressed genes were performed by DEseq with R. Genes with absolute log2 fold change > 1 were regarded as differentially expressed genes and *p* < 0.05 was used. Hierarchical clustering of log2-transformed FPKMs was generated by Cluster 3.0 and visualized by Java TreeView. The raw NGS data were deposited to the NCBI SRA database under Bioproject ID: PRJNA761186 (the accession numbers are from SRR15734269 to SRR15734277).

### CUT&Tag and sequencing

The eye and wing discs for the CUT&Tag assay were isolated from *w*^*1118*^, *Dmp18*^*d1*^, and *H2Av*^*810*^ third instar larvae. The CUT&Tag was performed using the Hyperactive In-Situ ChIP Library Prep Kit for Illumina (TD901, Vazyme, Nanjing, China). The antibodies for CUT&Tag assay: rabbit anti-H2Av (Active Motif, 39715), rabbit anti-H3K4me3 (Cell Signaling Technology, 9751), rabbit anti-H3K27ac (Cell Signaling Technology, 8173), and mouse anti-H3K27me3 (Abcam, ab6002). The sequencing was performed using the Illumina Novaseq 6000 platform. The raw NGS data were deposited to the NCBI SRA database under Bioproject ID: PRJNA761186 (the accession numbers are from SRR15734939 to SRR15734950).

## Supporting information

S1 Fig Knockdown of *Dmp18* in the eye causes eye defects. (A) Control. (B-C) Knockdown of *Dmp18* by RNAi induced adult eye defects.
(TIF)

S2 Fig The transcription of *Tfb5* is not affected in the *Dmp18* mutant. (A) The Schematic diagram of the genomic region of *Dmp18*, *Tfb5*, and *Dmp18*^*d1*^. The red arrows labeled the primer location for detecting *Tfb5* transcription. (B) Total RNA was extracted from control and homozygous *Dmp18*^*d1*^ larvae. The mRNA level of *Tfb5* was measured by RT-qPCR and normalized to *RPL32*. The mRNA level of *Tfb5* was not affected.
(TIF)

S3 Fig Loss of *Dmp18* does not affect the differentiation of photoreceptor cells. (A-A”) The differentiation of photoreceptor cells was not affected by *Dmp18* deletion. The Elav was expressed in the *Dmp18* mutant clones in the eye disc. GFP-free region marked the *Dmp18* mutant clones. (B-B”) The control eye disc staining with photoreceptor marker Elav. (C-C”) Homozygous *Dmp18*^*d1*^ eye disc staining with photoreceptor marker Elav. The photoreceptor cells showed normal Elav staining. (D-D”) Some homozygous *Dmp18*^*d1*^ eye disc showed an abnormal arrangement of photoreceptor cells. Genotypes: A-A”: *yw*, *hs-FLP; FRT*^*40A*^*-M(2L)-Ubi-GFP/FRT*^*40A*^*-Dmp18*^*d1*^; B-B”: *FRT*^*40A*^*/ FRT*^*40A*^; C-D”: *FRT*^*40A*^*-Dmp18*^*d1*^*/FRT*^*40A*^*-Dmp18*^*d1*^.
(TIF)

S4 Fig Loss of *Dmp18* induces apoptosis both in the eye and wing discs. (A-B”‘) TUNEL signals were increased in the *Dmp18* mutant clones generated by the *Minute* clone technique. GFP-free region marked the *Dmp18* mutant clones. (C-F’) TUNEL signals were increased in homozygous *Dmp18*^*d1*^ discs (E-E’ and F-F’) compared to the control discs (C-C’ and D-D’). Genotype: A-B”’: *yw*, *hs-FLP; FRT*^*40A*^*-M(2L)-Ubi-GFP/FRT*^*40A*^*-Dmp18*^*d1*^; C-D’: *FRT*^*40A*^*/FRT*^*40A*^; E-F’: *FRT*^*40A*^*-Dmp18*^*d1*^*/FRT*^*40A*^*-Dmp18*^*d1*^.
(TIF)

S5 Fig Over-expression of Dmp18-V5 inhibits *Dmp18* deletion-induced apoptosis. (A-B”) Loss of *Dmp18* activated Cas3* in the eye and wing discs. (C-D”’) Over-expression of Dmp18-V5 in the *Dmp18* mutant clones suppressed Cas3* activity in the eye (C-C”’) and wing (D-D”’) discs. GFP marked the mutant cells or mutant cells with over-expressed Dmp18-V5. Genotypes: A-B”: *yw*, *hs-FLP*, *tub-Gal4*, *UAS-nls-GFP/+; tub-Gal80*, *neoFRT*^*40A*^*/FRT*^*40A*^*-Dmp18*^*d1*^; C-D”’: *yw*, *hs-FLP*, *tub-Gal4*, *UAS-nls-GFP/+; tub-Gal80*, *neoFRT*^*40A*^*/FRT*^*40A*^*-Dmp18*^*d1*^*; UAS-Dmp18-V5/+*.
(TIF)

S6 Fig Bioinformatics analyses of Dmp18-regulated genes. (A) The volcano plot showed differentially expressed genes in homozygous *Dmp18*^*d1*^. 1266 genes were up-regulated, and 987 genes were down-regulated in *Dmp18*^*d1*^. The x-axis showed the log2 fold change and the y-axis showed corresponding -log10 *p* values. Red and blue dots marked differentially expressed genes (the absolute log2 fold change >1 and *p* <0.05, red dots indicated up-regulation and blue dots indicated down-regulation), and the gray dots marked no differentially expressed genes. (B) The Gene Ontology (GO) analysis of the Dmp18-regulated genes. The Dmp18-regulated genes were involved in multiple biological processes such as tissue development, metabolism, and stress response. (C) The KEGG pathway enrichment analysis of the Dmp18-regulated genes. The GO and KEGG enrichment analyses were performed by Metascape online (https://metascape.org) corresponding to the mini overlap was 3 and *p* <0.05.
(TIF)

S7 Fig Inhibition of JNK signaling does not suppress *Dmp18* deletion-induced apoptosis. (A) Loss of *Dmp18* activated Cas3* in the eye and wing discs. (B) Inhibition of JNK signaling by expressing dominant negative JNK (BSK^DN^) did not inhibit Cas3* activity in the *Dmp18* mutant clones in the eye and wing discs. (C) The Hid expression was up-regulated in the *Dmp18* mutant clones. (D-E) Expression of BSK^DN^ did not reduce the expression of Hid (D) and *hid-lacZ* (E). (F) Expression of BSK^DN^ suppressed the up-regulated *puc-lacZ* expression induced by *Dmp18* deletion in the eye and wing discs. GFP marked the mutant cells or the mutant cells with expressed UAS-BSK^DN^. Genotypes: A and C: *yw*, *hs-FLP*, *tub-Gal4*, *UAS-nls-GFP/+; tub-Gal80*, *neoFRT*^*40A*^*/FRT*^*40A*^*-Dmp18*^*d1*^; B and D: *yw*, *hs-FLP*, *tub-Gal4*, *UAS-nls-GFP/UAS-BSK*^*DN*^*; tub-Gal80*, *neoFRT*^*40A*^*/FRT*^*40A*^*-Dmp18*^*d1*^; E: *yw*, *hs-FLP*, *tub-Gal4*, *UAS-nls-GFP/UAS-BSK*^*DN*^*; tub-Gal80*, *neoFRT*^*40A*^*/FRT*^*40A*^*-Dmp18*^*d1*^*; hid-lacZ/+*; F: *yw*, *hs-FLP*, *tub-Gal4*, *UAS-nls-GFP/UAS-BSK*^*DN*^*; tub-Gal80*, *neoFRT*^*40A*^*/FRT*^*40A*^*-Dmp18*^*d1*^*; puc-lacZ/+*.
(TIF)

S8 Fig Knockdown of *p53* does not suppress *Dmp18* deletion-induced apoptosis. (A) Loss of *Dmp18* activated Cas3* in the eye and wing discs. (B) Knockdown of *p53* did not suppress Cas3* activity in the *Dmp18* mutant clones in the eye and wing discs. (C) Knockdown of *p53* did not reduce the up-regulated Hid expression in the wing disc. (D) The RT-qPCR results showed the transcription of *rpr*. The *rpr* still showed high expression when *p53* was knocked down by RNAi in the homozygous *Dmp18*^*d1*^. (E) The RT-qPCR results showed the knockdown efficiency of *p53* in the wild-type and homozygous *Dmp18*^*d1*^. The transcription of *p53* was reduced in the wild-type and *Dmp18*^*d1*^ when *p53* was knocked down by RNAi. The GFP marked mutant cells or mutant cells with knocking down *p53*. Genotypes: A: *yw*, *hs-FLP*, *tub-Gal4*, *UAS-nls-GFP/+; tub-Gal80*, *neoFRT*^*40A*^*/FRT*^*40A*^*-Dmp18*^*d1*^; B: *yw*, *hs-FLP*, *tub-Gal4*, *UAS-nls-GFP/+; tub-Gal80*, *neoFRT*^*40A*^*/FRT*^*40A*^*-Dmp18*^*d1*^*; UAS-p53 IR*^*5318*^*/+*; C: *yw*, *hs-FLP*, *tub-Gal4*, *UAS-nls-GFP/+; tub-Gal80*, *neoFRT*^*40A*^*/FRT*^*40A*^*-Dmp18*^*d1*^ and *yw*, *hs-FLP*, *tub-Gal4*, *UAS-nls-GFP/+; tub-Gal80*, *neoFRT*^*40A*^*/FRT*^*40A*^*-Dmp18*^*d1*^*; UAS-p53 IR*^*5318*^*/+*.
(TIF)

S9 Fig Bioinformatics analyses of the H2Av-regulated genes. (A)The volcano plot showed differentially expressed genes in homozygous *H2Av*^*810*^. 1365 genes were up-regulated, and 881 genes were down-regulated in *H2Av*^*810*^. The x-axis showed the log2 fold change and the y-axis showed corresponding -log10 *p* values. The red and blue dots marked differentially expressed genes (the absolute log2 fold change values>1 and *p*<0.05, red dots indicated up-regulated genes and blue dots indicated down-regulated genes), and the gray dots marked no differentially expressed genes. (B) The GO analysis of the H2Av-regulated genes. The H2Av-regulated genes were involved in multiple biologic processes including tissue development, metabolism, and stress response. (C) The KEGG pathway enrichment analysis of the H2Av-regulated genes. The GO and KEGG enrichment analyses were performed by Metascape online (https://metascape.org) corresponding to the mini overlap was 3 and *p* <0.05.
(TIF)

S10 Fig Bioinformatics analyses of the Dmp18 and H2Av co-regulated genes. Go and KEGG analyses showed that the Dmp18 and H2Av co-regulated genes were involved in multiple biological processes including tissue development, metabolism, and stress response. The GO and KEGG enrichment analyses were performed by Metascape online (https://metascape.org) corresponding to the mini overlap was 3 and *p* <0.05.
(TIF)

S11 Fig *H99* suppresses the apoptosis induced by *H2Av* knockdown. (A-A”) Control. (B-B”) TUNEL signals were increased when *H2Av* was knocked down by RNAi. (C-C”) *H99* suppressed the TUNEL signals induced by *H2Av* knockdown. Genotypes: A-A”: *ptc-gal4/+; UAS-luciferase IR/+*; B-B”: *ptc-gal4/UAS-H2Av IR*^*44056*^; C-C”: *ptc-gal4/UAS-H2Av IR*^*44056*^*; H99/+*.
(TIF)

S12 Fig Inhibition of JNK signaling does not suppress apoptosis induced by *H2Av* knockdown. (A-A”’) Expression of BSK^DN^ driven by *en-gal4* did not affect H2Av expression and TUNEL signals. (B-B”’) Expression of BSK^DN^ did not suppress the TUNEL signals induced by *H2Av* knockdown. (C-C’) The TUNEL signals were increased when *H2Av* was knocked down by RNAi. (D-D’) Knockdown of *H2Av* up-regulated *hid-lacZ* expression. (E-E”’) Expression of BSK^DN^ driven by *ptc-gal4* failed to suppress TUNEL signals (E’) and reduce the *hid-lacZ* expression (E”) in *H2Av* knockdown cells. (F-F”) Expression of BSK^DN^ driven by *ptc-gal4* suppressed the *puc-lacZ* expression induced by *H2Av* knockdown. Genotypes: A-A”’: *en-gal4*, *UAS-GFP/+; UAS-BSK*^*DN*^*/+*; B-B”’: *en-gal4*, *UAS-GFP/UAS-H2Av IR*^*44056*^*; UAS-BKS*^*DN*^*/*+; C-C’: *ptc-gal4/UAS-H2Av IR*^*44056*^. D-D’: *ptc-gal4/UAS-H2Av IR*^*44056*^; *hid-lacZ/+*; E-E”’: *ptc-gal4/UAS-H2Av IR*^*44056*^*; UAS-BSK*^*DN*^*/hid-lacZ*; F-F”: *ptc-gal4/UAS-H2Av IR*^*44056*^*; UAS-BSK*^*DN*^*/puc-lacZ*.
(TIF)

S13 Fig Knockdown of *p53* does not suppress apoptosis induced by *H2Av* knockdown. (A-A’) TUNEL signals were increased in *H2Av* knockdown cells. (B-C’) knockdown of *p53* did not reduce TUNEL signals induced by *H2Av* knockdown. (D-D’) Knockdown of *H2Av* increased *hid-lacZ* expression. (E-E’) Knockdown of *p53* did not suppress *hid-lacZ* expression induced by *H2Av* knockdown. (F) Statistical analysis of relative hid-lacZ fluorescence intensity in the D and E (the number of D = 17 and number of E = 15). To calculate the hid-lacZ fluorescence intensity, we subtracted the average fluorescence intensity near the ptc region (marked by a rectangle) from the average fluorescence intensity in the ptc region in the wing pouch. The data represented the relative hid-lacZ fluorescence intensity and were analyzed by Graphpad Prism 8 (San Diego, CA, USA), using the Mann Whitney test (means ± SEM). Genotypes: A-A’: *ptc-gal4/UAS-H2Av IR*^*44056*^; B-B’: *ptc-gal4/UAS-H2Av IR*^*44056*^*; UAS-p53 IR*^*5318*^*/+*; C-C’: *ptc-gal4/UAS-H2Av IR*^*44056*^*; UAS-p53 IR*^*2533*^*/+*; D-D’: *ptc-gal4/UAS-H2Av IR*^*44056*^*; hid-lacZ/+*; E-E”: *ptc-gal4/UAS-H2Av IR*^*44056*^*; UAS-p53 IR*^*5318*^/*hid-lacZ*.
(TIF)

S14 Fig Over-expression of H2Av-myc does not induce apoptosis in the wing disc. (A-A”) Control. (B-B”’) Over-expression of H2Av-myc driven by *en-gal4* did not activate Cas3* in the posterior region of wing disc.
(TIF)

S15 Fig The commercial H2Av antibody detects the binding of H2Av at the polytene chromosome in homozygous *H2Av*^*810*^. (A-B’) The H2Av expression level was reduced in the *H2Av* mutant clones in the eye and wing discs. The GFP marked the *H2Av* mutant clones. (C-C’) The commercial H2Av antibody detected the binding of H2Av at the polytene chromosome in homozygous *H2Av*^*810*^. (D-D’) The commercial H2Av antibody detected the H2Av staining in some salivary gland cells in homozygous *H2Av*^*810*^. Genotypes: A-B’: *yw*, *hs-FLP*, *UAS-GFP/+; tub-Gal4*, *FRT*^*82B*^, *tub-Gal80/FRT*^*82B*^*-H2Av*^*810*^; C-D’: *H2Av*^*810*^*/H2Av*^*810*^.
(TIF)

S16 Fig The RT-qPCR analysis of the transcription changes of *hid* and *rpr* in the single homozygous *Dmp18*^*d1*^ larva. (A) Compared with control 1, none of the four homozygous *Dmp18*^*d1*^ larvae showed increased *hid* transcription. (B-C) Compared with control 2 and control 3, two of the four homozygous *Dmp18*^*d1*^ larvae showed increased *hid* transcription. (D) Compared with control 4, none of the four homozygous *Dmp18*^*d1*^ larvae showed increased *hid* transcription. Except for the mutant 3, which did not show an obvious change in *rpr* transcription compared with control 4 (the fold change is 1.6), all other *Dmp18* mutant larvae showed increased *rpr* transcription compared with controls. (The fold change >2 was considered up-regulated).
(TIF)

S1 Table The primers for RT-qPCR. (DOCX)

S2 Table The list of Dmp18-regulated genes (DEGs) from RNA-seq. (XLSX)

S3 Table The list of H2Av-regulated genes (DEGs) from RNA-seq. (XLSX)
