# Supplementary figures and images for: Chromatin remodeler Dmp18 regulates apoptosis by controlling H2Av incorporation in Drosophila imaginal disc development
Source: PLoS Genet. 2022 Sep 27;18(9):e1010395. doi: 10.1371/journal.pgen.1010395 (PMC9514664; doi:10.1371/journal.pgen.1010395)

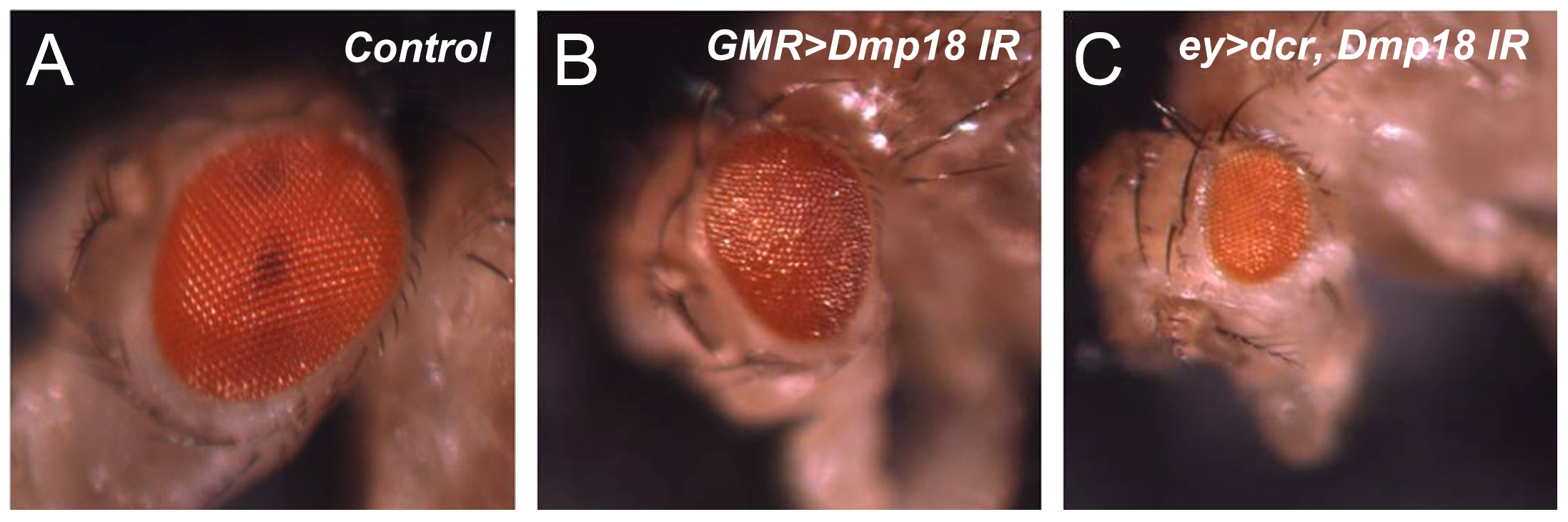

Supplement: S1 Fig — (A) Control. (B-C) Knockdown of Dmp18 by RNAi induced adult eye defects. (TIF) [file pgen.1010395.s001.tif]

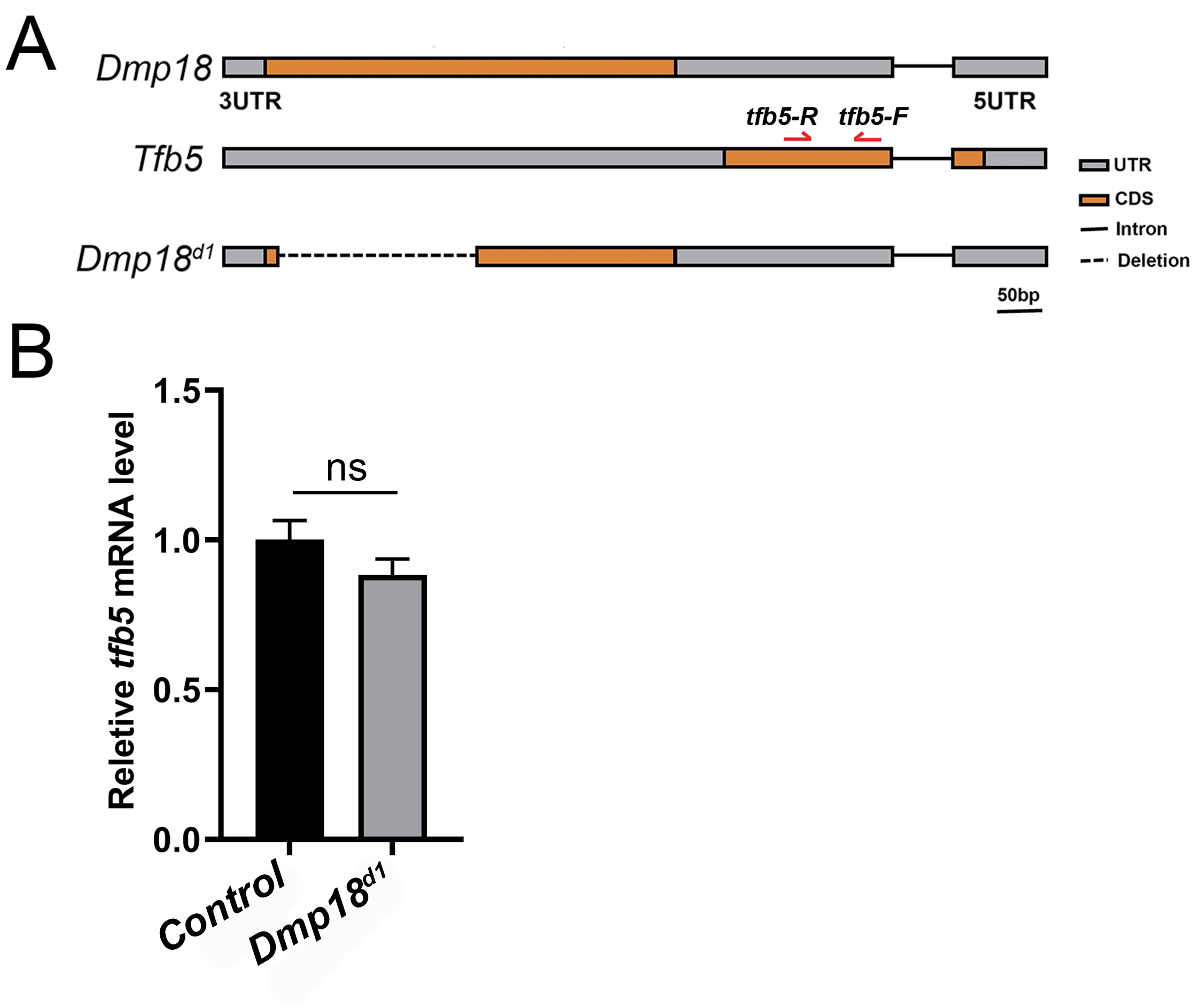

Supplement: S2 Fig — (A) The Schematic diagram of the genomic region of Dmp18, Tfb5, and Dmp18d1. The red arrows labeled the primer location for detecting Tfb5 transcription. (B) Total RNA was extracted from control and homozygous Dmp18d1 larvae. The mRNA level of Tfb5 was measured by RT-qPCR and normalized to RPL32. The mRNA level of Tfb5 was not affected. (TIF) [file pgen.1010395.s002.tif]

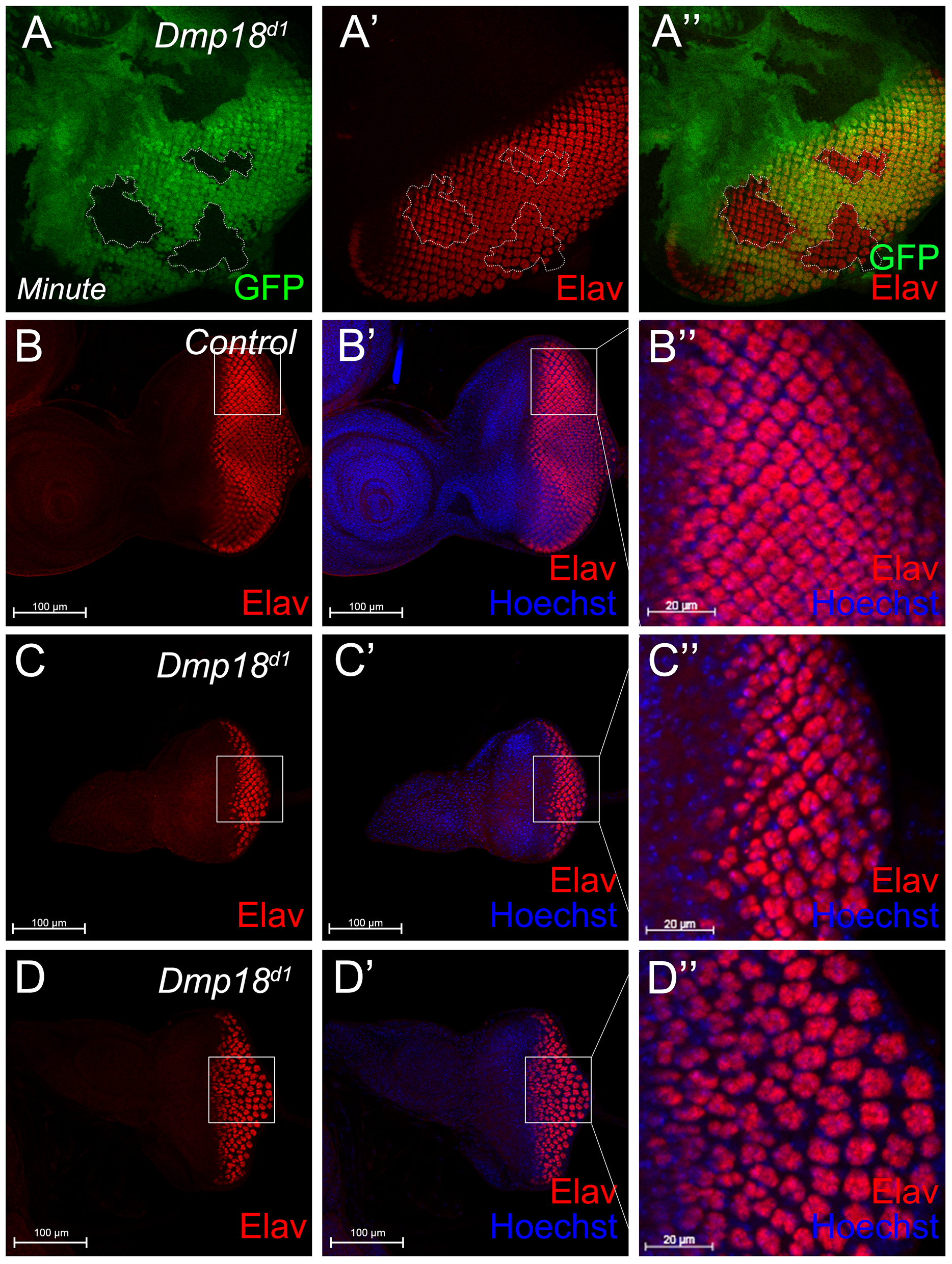

Supplement: S3 Fig — (A-A”) The differentiation of photoreceptor cells was not affected by Dmp18 deletion. The Elav was expressed in the Dmp18 mutant clones in the eye disc. GFP-free region marked the Dmp18 mutant clones. (B-B”) The control eye disc staining with photoreceptor marker Elav. (C-C”) Homozygous Dmp18d1 eye disc staining with photoreceptor marker Elav. The photoreceptor cells showed normal Elav staining. (D-D”) Some homozygous Dmp18d1 eye disc showed an abnormal arrangement of photoreceptor cells. Genotypes: A-A”: yw, hs-FLP; FRT40A-M(2L)-Ubi-GFP/FRT40A-Dmp18d1; B-B”: FRT40A/ FRT40A; C-D”: FRT40A-Dmp18d1/FRT40A-Dmp18d1. (TIF) [file pgen.1010395.s003.tif]

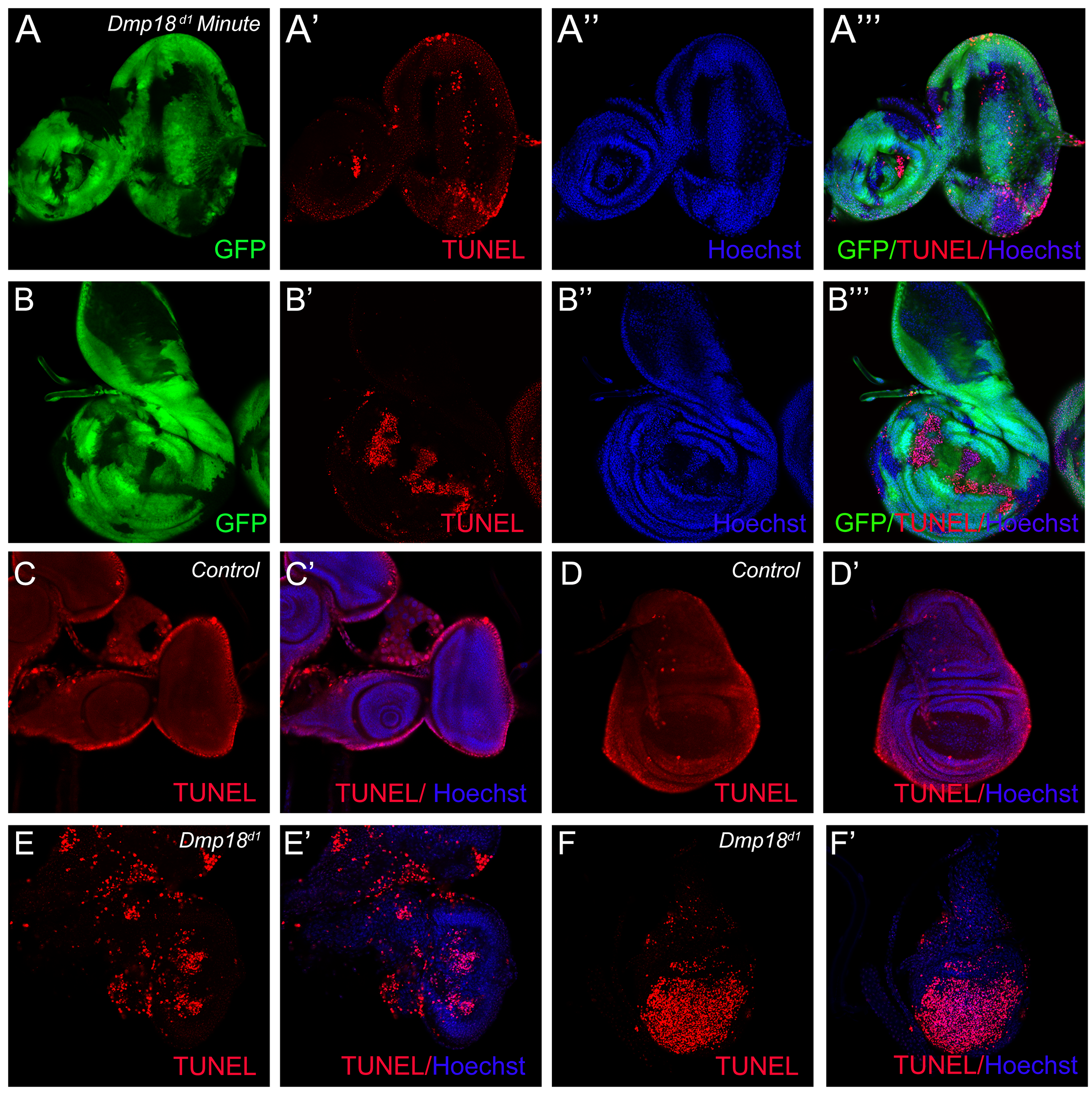

Supplement: S4 Fig — (A-B”‘) TUNEL signals were increased in the Dmp18 mutant clones generated by the Minute clone technique. GFP-free region marked the Dmp18 mutant clones. (C-F’) TUNEL signals were increased in homozygous Dmp18d1 discs (E-E’ and F-F’) compared to the control discs (C-C’ and D-D’). Genotype: A-B”’: yw, hs-FLP; FRT40A-M(2L)-Ubi-GFP/FRT40A-Dmp18d1; C-D’: FRT40A/FRT40A; E-F’: FRT40A-Dmp18d1/FRT40A-Dmp18d1. (TIF) [file pgen.1010395.s004.tif]

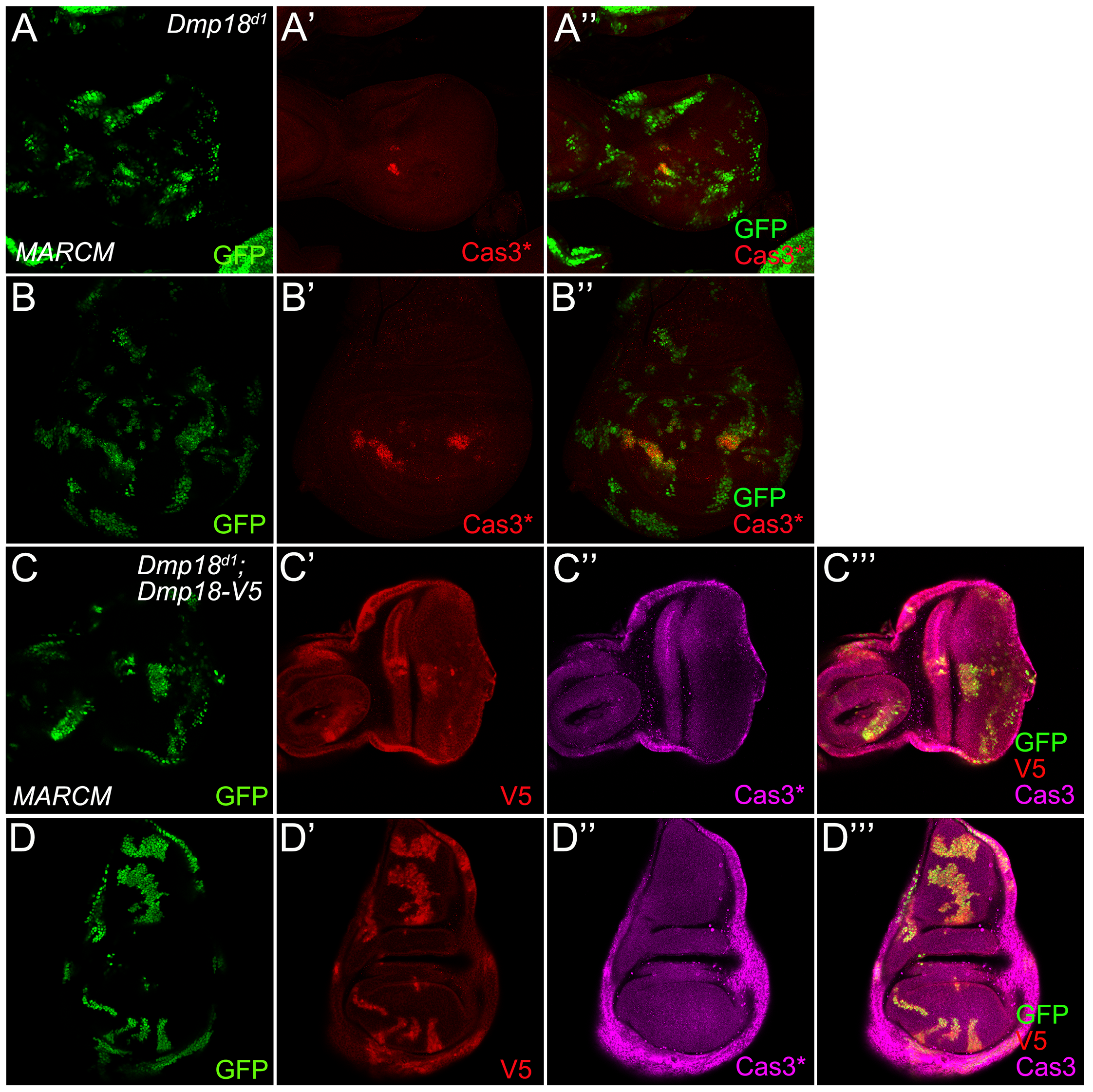

Supplement: S5 Fig — (A-B”) Loss of Dmp18 activated Cas3* in the eye and wing discs. (C-D”’) Over-expression of Dmp18-V5 in the Dmp18 mutant clones suppressed Cas3* activity in the eye (C-C”’) and wing (D-D”’) discs. GFP marked the mutant cells or mutant cells with over-expressed Dmp18-V5. Genotypes: A-B”: yw, hs-FLP, tub-Gal4, UAS-nls-GFP/+; tub-Gal80, neoFRT40A/FRT40A-Dmp18d1; C-D”’: yw, hs-FLP, tub-Gal4, UAS-nls-GFP/+; tub-Gal80, neoFRT40A/FRT40A-Dmp18d1; UAS-Dmp18-V5/+. (TIF) [file pgen.1010395.s005.tif]

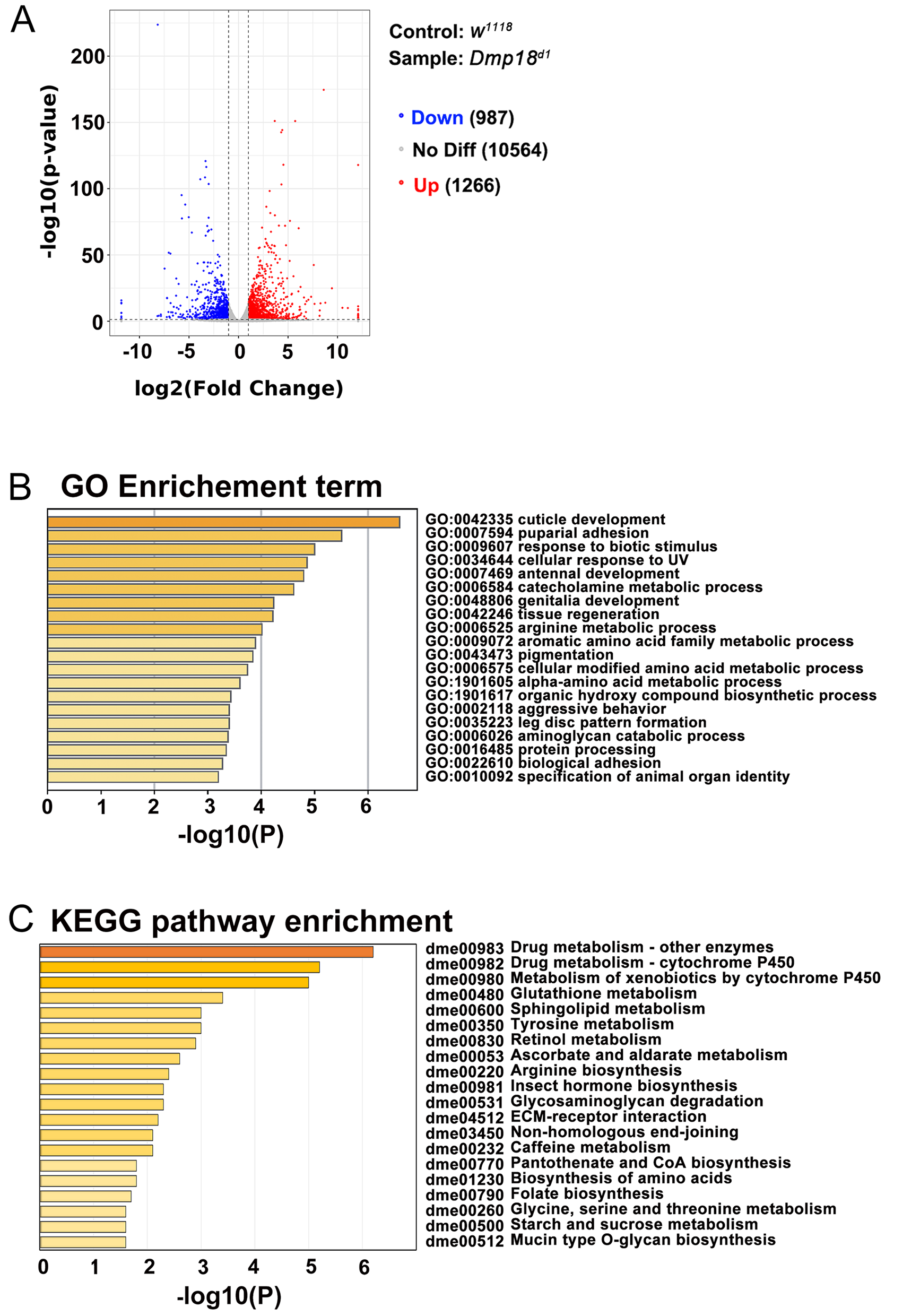

Supplement: S6 Fig — (A) The volcano plot showed differentially expressed genes in homozygous Dmp18d1. 1266 genes were up-regulated, and 987 genes were down-regulated in Dmp18d1. The x-axis showed the log2 fold change and the y-axis showed corresponding -log10 p values. Red and blue dots marked differentially expressed genes (the absolute log2 fold change >1 and p <0.05, red dots indicated up-regulation and blue dots indicated down-regulation), and the gray dots marked no differentially expressed genes. (B) The Gene Ontology (GO) analysis of the Dmp18-regulated genes. The Dmp18-regulated genes were involved in multiple biological processes such as tissue development, metabolism, and stress response. (C) The KEGG pathway enrichment analysis of the Dmp18-regulated genes. The GO and KEGG enrichment analyses were performed by Metascape online (https://metascape.org) corresponding to the mini overlap was 3 and p <0.05. (TIF) [file pgen.1010395.s006.tif]

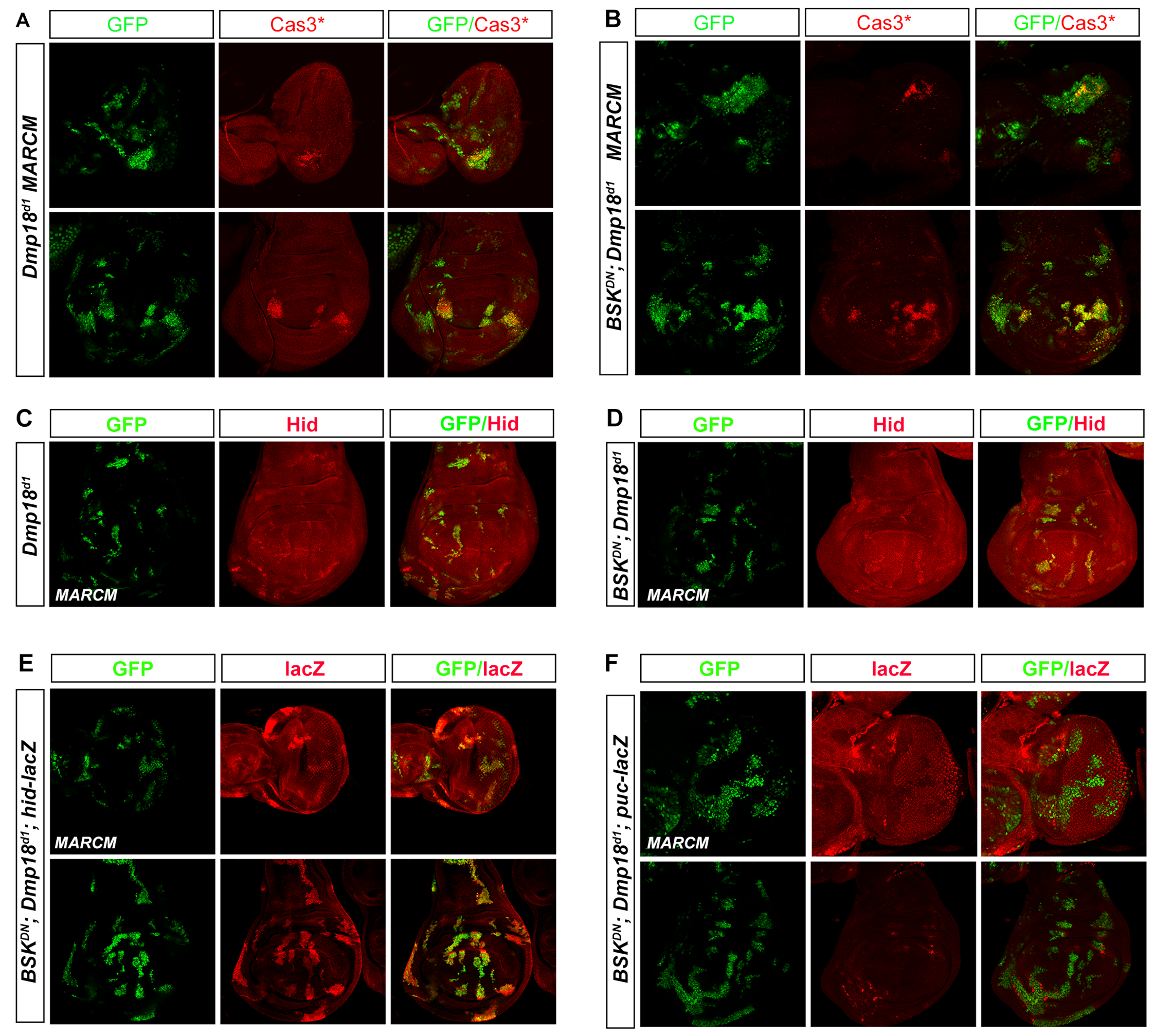

Supplement: S7 Fig — (A) Loss of Dmp18 activated Cas3* in the eye and wing discs. (B) Inhibition of JNK signaling by expressing dominant negative JNK (BSKDN) did not inhibit Cas3* activity in the Dmp18 mutant clones in the eye and wing discs. (C) The Hid expression was up-regulated in the Dmp18 mutant clones. (D-E) Expression of BSKDN did not reduce the expression of Hid (D) and hid-lacZ (E). (F) Expression of BSKDN suppressed the up-regulated puc-lacZ expression induced by Dmp18 deletion in the eye and wing discs. GFP marked the mutant cells or the mutant cells with expressed UAS-BSKDN. Genotypes: A and C: yw, hs-FLP, tub-Gal4, UAS-nls-GFP/+; tub-Gal80, neoFRT40A/FRT40A-Dmp18d1; B and D: yw, hs-FLP, tub-Gal4, UAS-nls-GFP/UAS-BSKDN; tub-Gal80, neoFRT40A/FRT40A-Dmp18d1; E: yw, hs-FLP, tub-Gal4, UAS-nls-GFP/UAS-BSKDN; tub-Gal80, neoFRT40A/FRT40A-Dmp18d1; hid-lacZ/+; F: yw, hs-FLP, tub-Gal4, UAS-nls-GFP/UAS-BSKDN; tub-Gal80, neoFRT40A/FRT40A-Dmp18d1; puc-lacZ/+. (TIF) [file pgen.1010395.s007.tif]

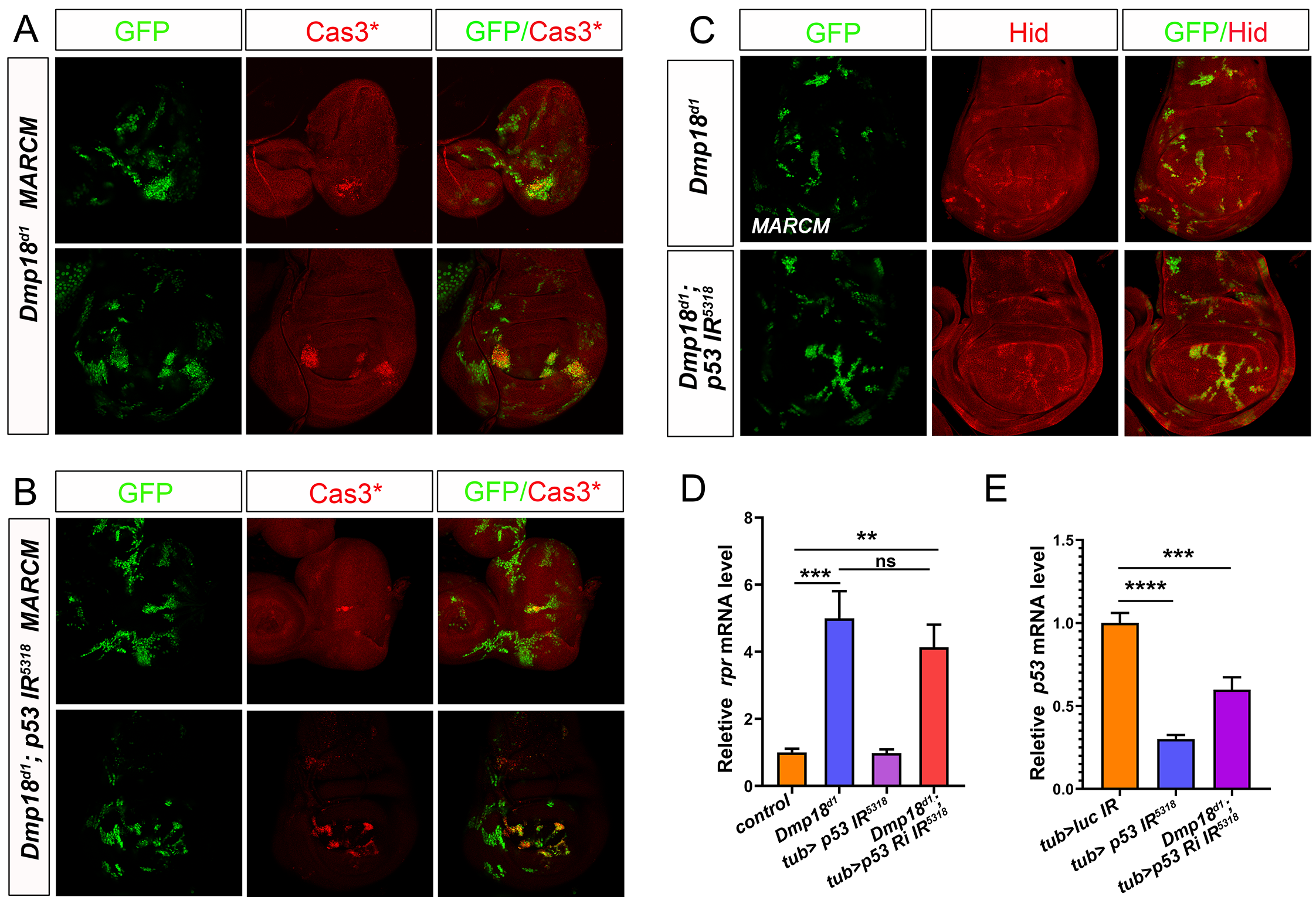

Supplement: S8 Fig — (A) Loss of Dmp18 activated Cas3* in the eye and wing discs. (B) Knockdown of p53 did not suppress Cas3* activity in the Dmp18 mutant clones in the eye and wing discs. (C) Knockdown of p53 did not reduce the up-regulated Hid expression in the wing disc. (D) The RT-qPCR results showed the transcription of rpr. The rpr still showed high expression when p53 was knocked down by RNAi in the homozygous Dmp18d1. (E) The RT-qPCR results showed the knockdown efficiency of p53 in the wild-type and homozygous Dmp18d1. The transcription of p53 was reduced in the wild-type and Dmp18d1 when p53 was knocked down by RNAi. The GFP marked mutant cells or mutant cells with knocking down p53. Genotypes: A: yw, hs-FLP, tub-Gal4, UAS-nls-GFP/+; tub-Gal80, neoFRT40A/FRT40A-Dmp18d1; B: yw, hs-FLP, tub-Gal4, UAS-nls-GFP/+; tub-Gal80, neoFRT40A/FRT40A-Dmp18d1; UAS-p53 IR5318/+; C: yw, hs-FLP, tub-Gal4, UAS-nls-GFP/+; tub-Gal80, neoFRT40A/FRT40A-Dmp18d1 and yw, hs-FLP, tub-Gal4, UAS-nls-GFP/+; tub-Gal80, neoFRT40A/FRT40A-Dmp18d1; UAS-p53 IR5318/+. (TIF) [file pgen.1010395.s008.tif]

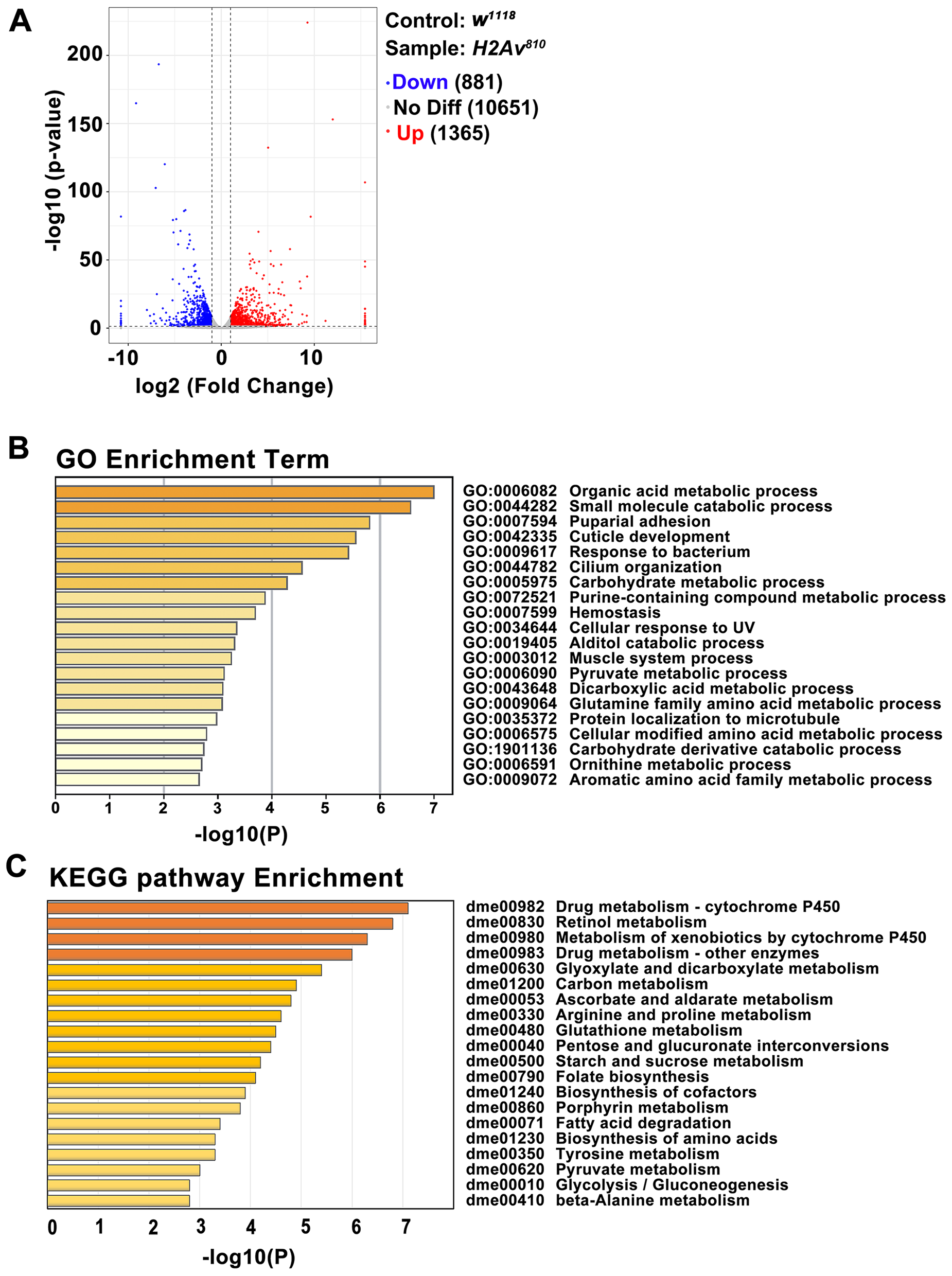

Supplement: S9 Fig — (A)The volcano plot showed differentially expressed genes in homozygous H2Av810. 1365 genes were up-regulated, and 881 genes were down-regulated in H2Av810. The x-axis showed the log2 fold change and the y-axis showed corresponding -log10 p values. The red and blue dots marked differentially expressed genes (the absolute log2 fold change values>1 and p<0.05, red dots indicated up-regulated genes and blue dots indicated down-regulated genes), and the gray dots marked no differentially expressed genes. (B) The GO analysis of the H2Av-regulated genes. The H2Av-regulated genes were involved in multiple biologic processes including tissue development, metabolism, and stress response. (C) The KEGG pathway enrichment analysis of the H2Av-regulated genes. The GO and KEGG enrichment analyses were performed by Metascape online (https://metascape.org) corresponding to the mini overlap was 3 and p <0.05. (TIF) [file pgen.1010395.s009.tif]

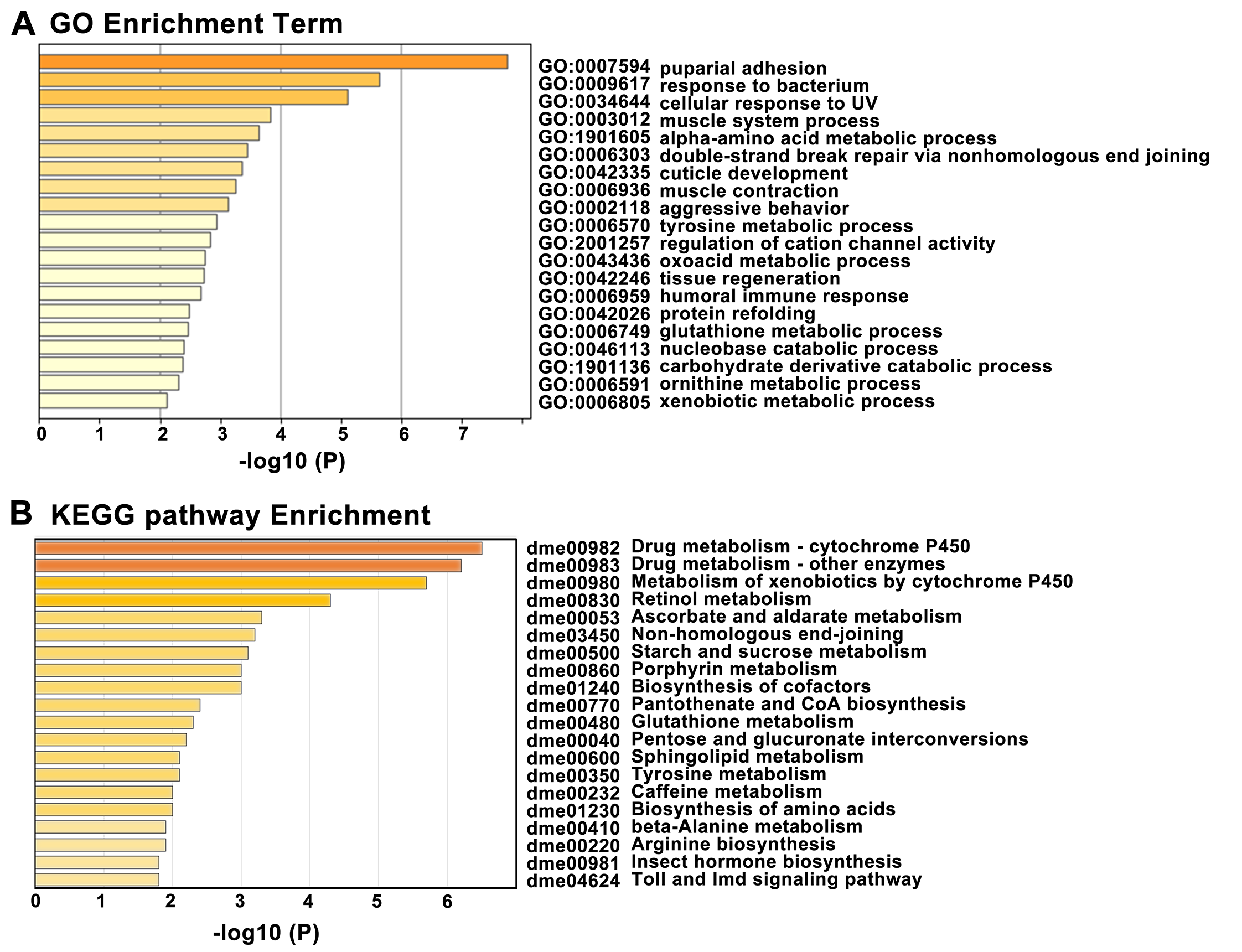

Supplement: S10 Fig — Go and KEGG analyses showed that the Dmp18 and H2Av co-regulated genes were involved in multiple biological processes including tissue development, metabolism, and stress response. The GO and KEGG enrichment analyses were performed by Metascape online (https://metascape.org) corresponding to the mini overlap was 3 and p <0.05. (TIF) [file pgen.1010395.s010.tif]

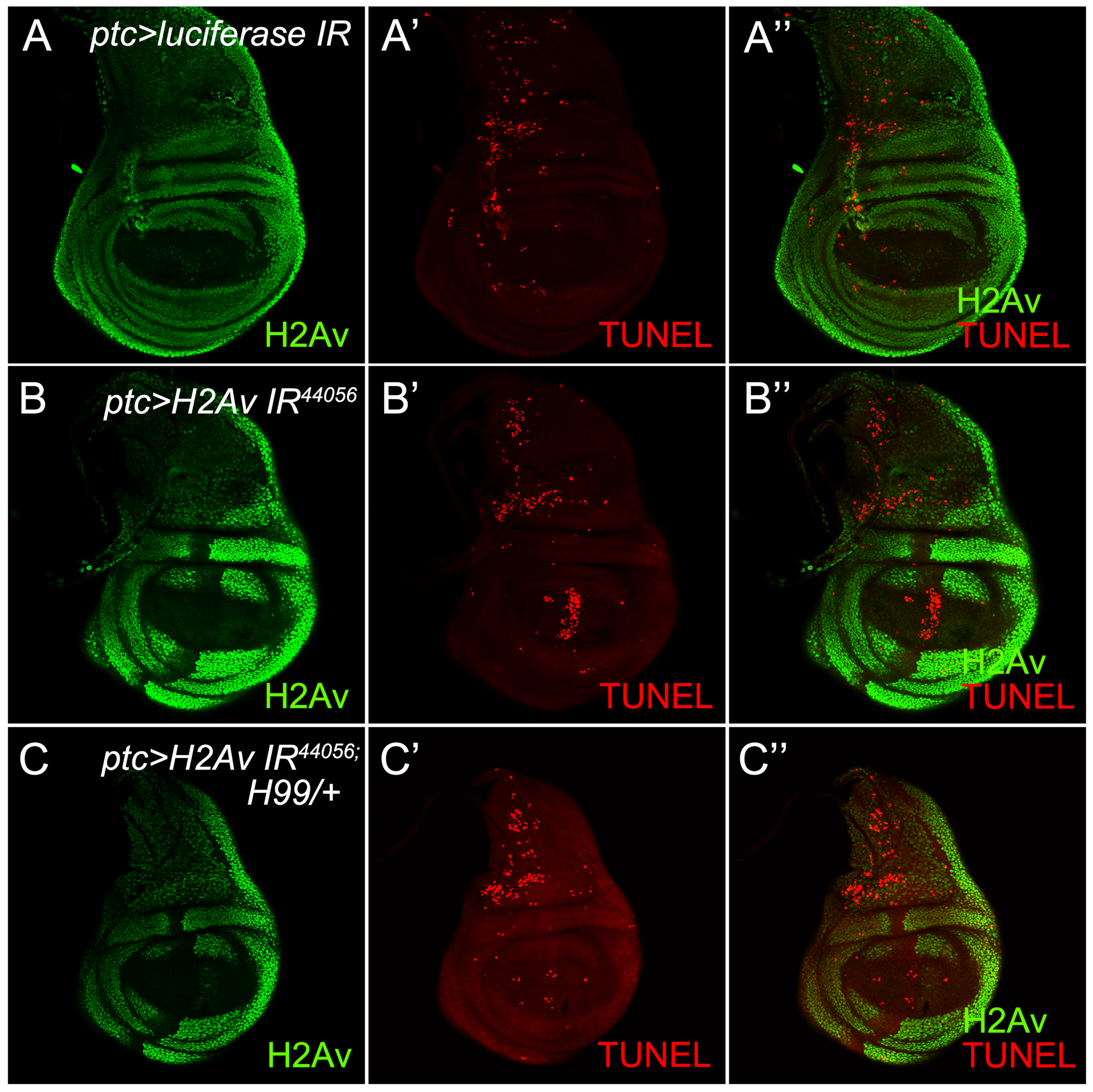

Supplement: S11 Fig — (A-A”) Control. (B-B”) TUNEL signals were increased when H2Av was knocked down by RNAi. (C-C”) H99 suppressed the TUNEL signals induced by H2Av knockdown. Genotypes: A-A”: ptc-gal4/+; UAS-luciferase IR/+; B-B”: ptc-gal4/UAS-H2Av IR44056; C-C”: ptc-gal4/UAS-H2Av IR44056; H99/+. (TIF) [file pgen.1010395.s011.tif]

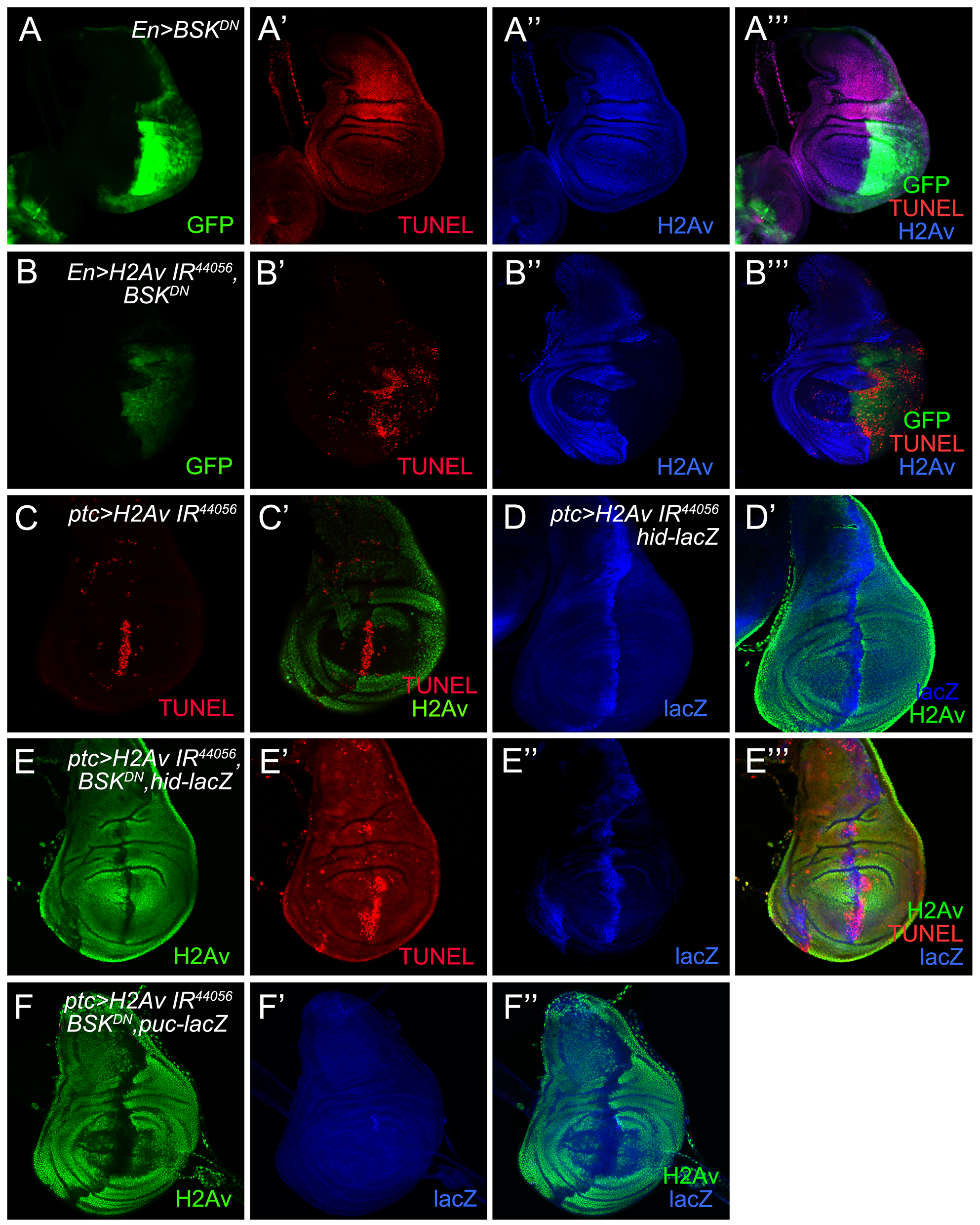

Supplement: S12 Fig — (A-A”’) Expression of BSKDN driven by en-gal4 did not affect H2Av expression and TUNEL signals. (B-B”’) Expression of BSKDN did not suppress the TUNEL signals induced by H2Av knockdown. (C-C’) The TUNEL signals were increased when H2Av was knocked down by RNAi. (D-D’) Knockdown of H2Av up-regulated hid-lacZ expression. (E-E”’) Expression of BSKDN driven by ptc-gal4 failed to suppress TUNEL signals (E’) and reduce the hid-lacZ expression (E”) in H2Av knockdown cells. (F-F”) Expression of BSKDN driven by ptc-gal4 suppressed the puc-lacZ expression induced by H2Av knockdown. Genotypes: A-A”’: en-gal4, UAS-GFP/+; UAS-BSKDN/+; B-B”’: en-gal4, UAS-GFP/UAS-H2Av IR44056; UAS-BKSDN/+; C-C’: ptc-gal4/UAS-H2Av IR44056. D-D’: ptc-gal4/UAS-H2Av IR44056; hid-lacZ/+; E-E”’: ptc-gal4/UAS-H2Av IR44056; UAS-BSKDN/hid-lacZ; F-F”: ptc-gal4/UAS-H2Av IR44056; UAS-BSKDN/puc-lacZ. (TIF) [file pgen.1010395.s012.tif]

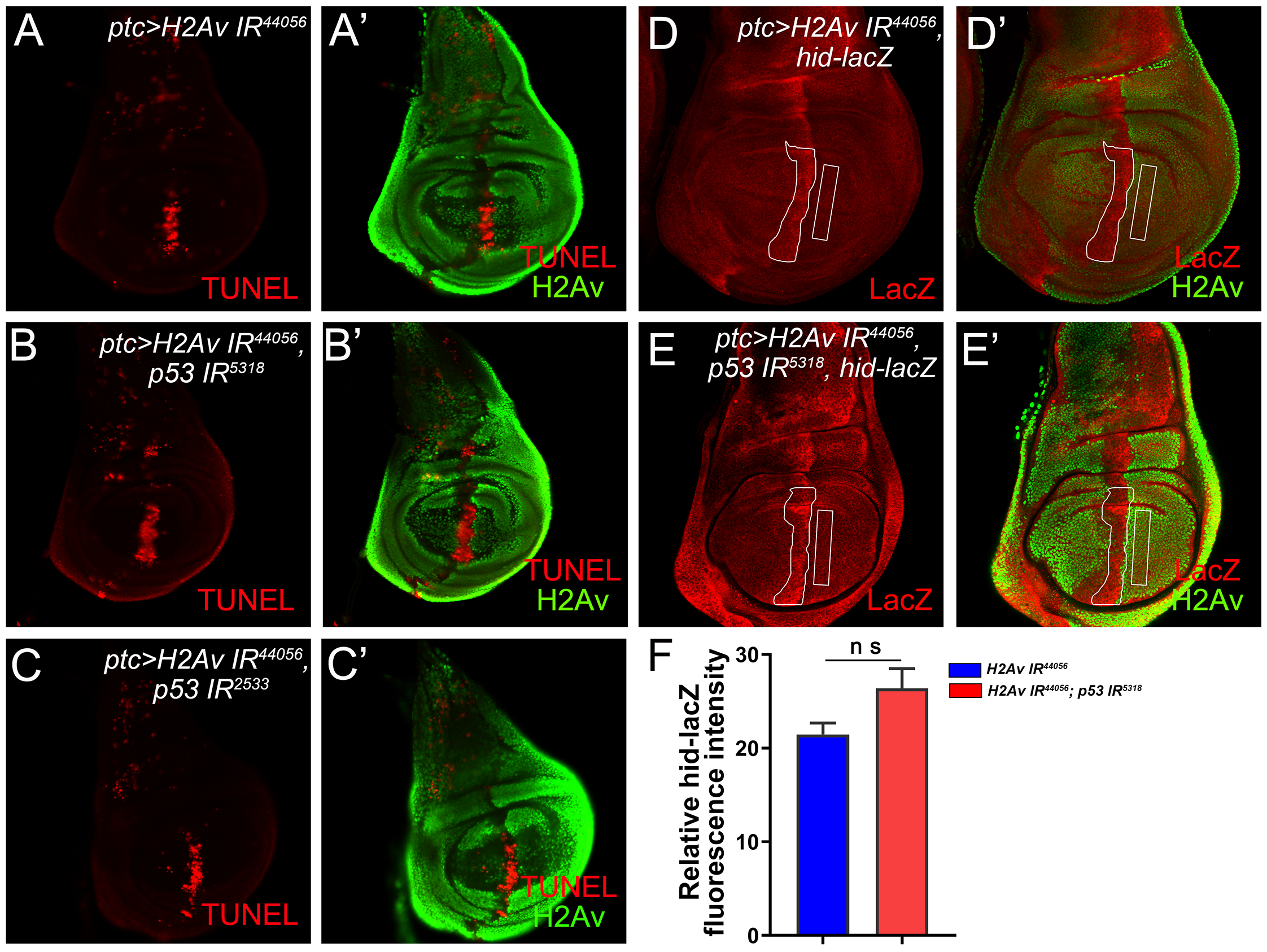

Supplement: S13 Fig — (A-A’) TUNEL signals were increased in H2Av knockdown cells. (B-C’) knockdown of p53 did not reduce TUNEL signals induced by H2Av knockdown. (D-D’) Knockdown of H2Av increased hid-lacZ expression. (E-E’) Knockdown of p53 did not suppress hid-lacZ expression induced by H2Av knockdown. (F) Statistical analysis of relative hid-lacZ fluorescence intensity in the D and E (the number of D = 17 and number of E = 15). To calculate the hid-lacZ fluorescence intensity, we subtracted the average fluorescence intensity near the ptc region (marked by a rectangle) from the average fluorescence intensity in the ptc region in the wing pouch. The data represented the relative hid-lacZ fluorescence intensity and were analyzed by Graphpad Prism 8 (San Diego, CA, USA), using the Mann Whitney test (means ± SEM). Genotypes: A-A’: ptc-gal4/UAS-H2Av IR44056; B-B’: ptc-gal4/UAS-H2Av IR44056; UAS-p53 IR5318/+; C-C’: ptc-gal4/UAS-H2Av IR44056; UAS-p53 IR2533/+; D-D’: ptc-gal4/UAS-H2Av IR44056; hid-lacZ/+; E-E”: ptc-gal4/UAS-H2Av IR44056; UAS-p53 IR5318/hid-lacZ. (TIF) [file pgen.1010395.s013.tif]

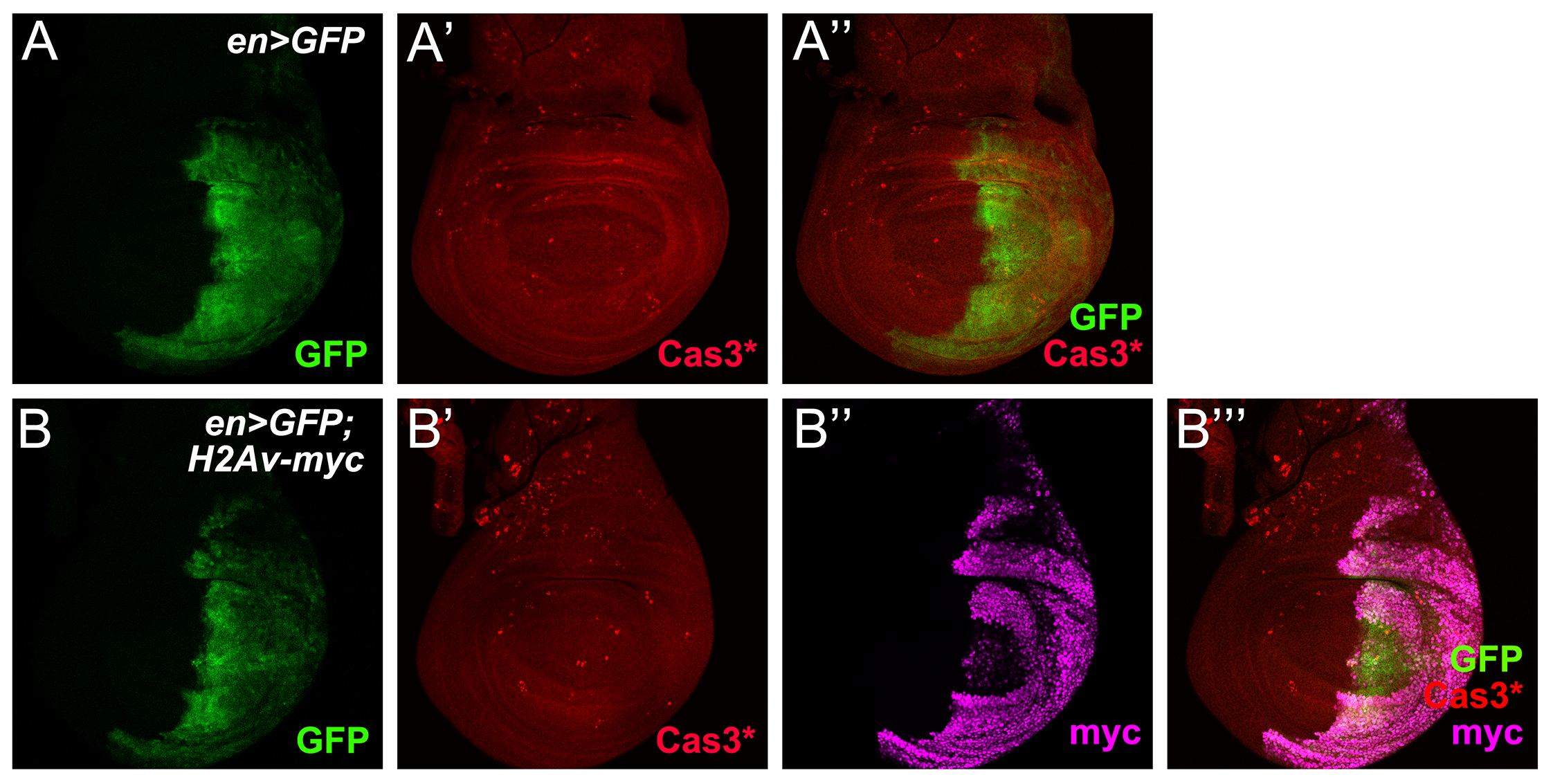

Supplement: S14 Fig — (A-A”) Control. (B-B”’) Over-expression of H2Av-myc driven by en-gal4 did not activate Cas3* in the posterior region of wing disc. (TIF) [file pgen.1010395.s014.tif]

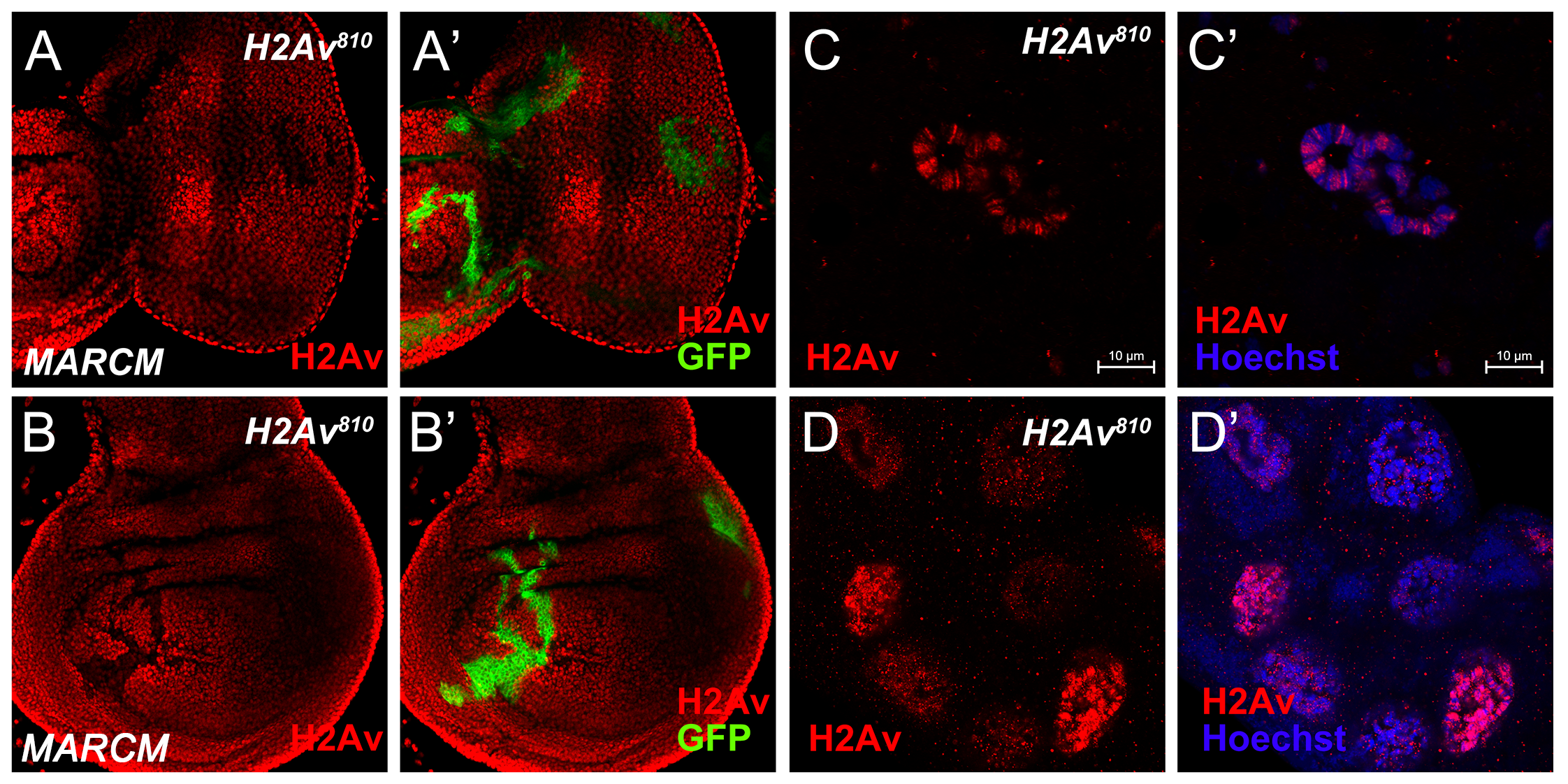

Supplement: S15 Fig — (A-B’) The H2Av expression level was reduced in the H2Av mutant clones in the eye and wing discs. The GFP marked the H2Av mutant clones. (C-C’) The commercial H2Av antibody detected the binding of H2Av at the polytene chromosome in homozygous H2Av810. (D-D’) The commercial H2Av antibody detected the H2Av staining in some salivary gland cells in homozygous H2Av810. Genotypes: A-B’: yw, hs-FLP, UAS-GFP/+; tub-Gal4, FRT82B, tub-Gal80/FRT82B-H2Av810; C-D’: H2Av810/H2Av810. (TIF) [file pgen.1010395.s015.tif]

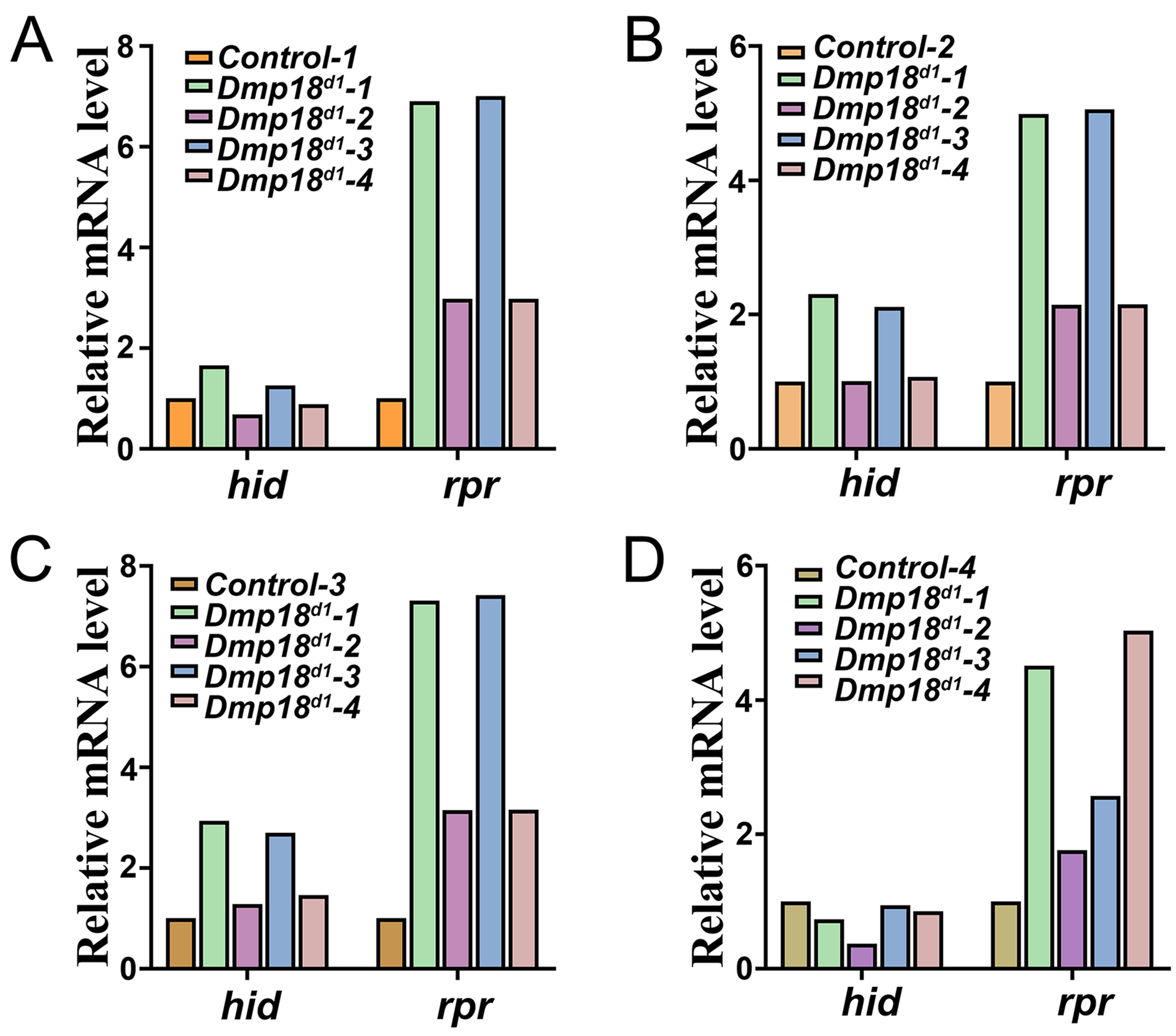

Supplement: S16 Fig — (A) Compared with control 1, none of the four homozygous Dmp18d1 larvae showed increased hid transcription. (B-C) Compared with control 2 and control 3, two of the four homozygous Dmp18d1 larvae showed increased hid transcription. (D) Compared with control 4, none of the four homozygous Dmp18d1 larvae showed increased hid transcription. Except for the mutant 3, which did not show an obvious change in rpr transcription compared with control 4 (the fold change is 1.6), all other Dmp18 mutant larvae showed increased rpr transcription compared with controls. (The fold change >2 was considered up-regulated). (TIF) [file pgen.1010395.s016.tif]
