# Supplementary material for: Chromatin remodeler Dmp18 regulates apoptosis by controlling H2Av incorporation in Drosophila imaginal disc development
Source: PLoS Genet. 2022 Sep 27;18(9):e1010395. doi: 10.1371/journal.pgen.1010395 (PMC9514664; doi:10.1371/journal.pgen.1010395)
Supplement: S1 Table — (DOCX) [file pgen.1010395.s017.docx]

**Supplementary Table 1. Primers used for RT-qPCR**

| Primers | Sequence |
| --- | --- |
| *H2Av*-forward | 5’-CGCACTACGTCACATGGAC-3’ |
| *H2Av*-reverse | 5’-TGGCGAGGAGTGATACGTTTC-3’ |
| *Tfb5*-forward | 5’-CTGCTGCACTTGGACGAAAA-3’ |
| *Tfb5*-reverse | 5’-CGCCTGCAAAACTTCCACAA-3’ |
| *rpr*-forward | 5’-AATAGTCCAGTCCAAAATCCAGAGT-3’ |
| *rpr*-reverse | 5’-TAGAAACAAAACCATTATCACAATCG-3’ |
| *hid*-forward | 5’-CACCGACCAAGTGCTATACG-3’ |
| *hid*-reverse | 5’-GGCGGATACTGGAAGATTTGC-3’ |
| *grim*-forward | 5’-GGAGTTTGGATGCTGGGATCT-3’ |
| *grim*-reverse | 5’-GTCCTCATCGTTGTTCTGACC-3’ |
| *skl*-forward | 5’- GACTTTGAAGGACCTCCGTCTG-3’ |
| *skl*-reverse | 5’- CGCCTGCGGTTGTATTTGA-3’ |
| *Diap1*-forward | 5’-CCCAGTATCCCGAATACGCC-3’ |
| *Diap1*-reverse | 5’-TCTGTTTCAGGTTCCTCGGC-3’ |
| *Diap2*-forward | 5’- CCACCAGCAAGGCAGCATC -3’ |
| *Diap2*-reverse | 5’- GCCACAGGGCAGGAACACT -3’ |
| *Debcl*-forward | 5’- TACAGTGCCTGATTGACGGT-3’ |
| *Debcl*-reverse | 5’- AGCAGCGAATACAGTTGACC-3’ |
| *Dronc*-forward | 5’- TGGACGAGAAGGATGTGCG -3’ |
| *Dronc*-reverse | 5’- TTAGCGAGATAAAGGGTGGC -3’ |
| *Dcp-1*-forward | 5’-ACTACTTCACTGCCACCTTCTGC-3’ |
| *Dcp-1*-reverse | 5’-TCGGCGTGTATGGGTATCTTG-3’ |
| *Drice*-forward | 5’- GGCTGGACATCCTGACCCT -3’ |
| *Drice*-reverse | 5’- CCAACTGCTTGTCGCTGAAAC -3’ |
| *puc*-roward | 5’-CAACCCCGCACCTGAATAGTC-3’ |
| *puc*-reverse | 5’-CCACATCATCGTAATCAAACCC-3’ |
| *p53*-forward | 5’- CTTTGACGGCCAATAACGCA-3’ |
| *p53*-reverse | 5’-TTCAGGGGGACTACAACGGA-3’ |
| *RPL32*-forward | 5’-AAGCACTTCATCCGCCACC-3’ |
| *RPL32*-reverse | 5’-CGATCTCGCCGCAGTAAACG-3’ |
